# Graft-derived neurons and bystander effects are maintained for six months after human iPSC-derived NESC transplantation in mice’s cerebella

**DOI:** 10.1038/s41598-024-53542-x

**Published:** 2024-02-08

**Authors:** Liliana S. Mendonça, Daniel Henriques, Vanessa Fernandes, Ricardo Moreira, João Brás, Sónia Duarte, Jens C. Schwamborn, Luís Pereira de Almeida

**Affiliations:** 1 Center for Neuroscience and Cell Biology, University of Coimbra, Coimbra, Portugal; 2 Center for Innovative Biomedicine and Biotechnology, University of Coimbra, Coimbra, Portugal; 3 Institute of Interdisciplinary Research, University of Coimbra, Coimbra, Portugal; 4 Faculty of Pharmacy, University of Coimbra, Coimbra, Portugal; 5 Luxembourg Centre for Systems Biomedicine, University of Luxembourg, Belvaux, Luxembourg

**Keywords:** Spinocerebellar ataxia, Stem-cell biotechnology, Induced pluripotent stem cells, Regeneration, Regeneration and repair in the nervous system, Stem cells in the nervous system

## Abstract

Machado-Joseph disease (MJD) is a neurodegenerative disorder characterized by widespread neuronal death affecting the cerebellum. Cell therapy can trigger neuronal replacement and neuroprotection through bystander effects providing a therapeutic option for neurodegenerative diseases. Here, human control (CNT) and MJD iPSC-derived neuroepithelial stem cells (NESC) were established and tested for their therapeutic potential. Cells’ neuroectodermal phenotype was demonstrated. Brain organoids obtained from the Control NESC showed higher mRNA levels of genes related to stem cells' bystander effects, such as BDNF, NEUROD1, and NOTCH1, as compared with organoids produced from MJD NESC, suggesting that Control NESC have a higher therapeutic potential. Graft-derived glia and neurons, such as cells positive for markers of cerebellar neurons, were detected six months after NESC transplantation in mice cerebella. The graft-derived neurons established excitatory and inhibitory synapses in the host cerebella, although CNT neurons exhibited higher excitatory synapse numbers compared with MJD neurons. Cell grafts, mainly CNT NESC, sustained the bystander effects through modulation of inflammatory interleukins (IL1B and IL10), neurotrophic factors (NGF), and neurogenesis-related proteins (Msi1 and NeuroD1), for six months in the mice cerebella. Altogether this study demonstrates the long-lasting therapeutic potential of human iPSC-derived NESC in the cerebellum.

## Introduction

Machado-Joseph disease (MJD) is a neurodegenerative disease caused by an abnormally expanded polyglutamine tract in the ataxin-3 protein. This mutant ataxin-3 causes neuronal degeneration in numerous brain regions and the cerebellum is one of the most affected of them^1–3^. Patients exhibit severe motor coordination problems, such as dysarthria and ataxia, and non-motor symptoms, namely cognitive impairments and sleep disorders^1,2,4^. There is no treatment for this disease and by the time of the diagnosis patients already display widespread neuronal death. Therefore, MJD patients might significantly benefit from cell-based therapies promoting cell replacement of lost neurons and neuroprotection through bystander effects^5–10^.

We have previously demonstrated that the transplantation of murine neural stem cells (NSC) into the cerebellum of adult MJD transgenic mice reduces the neuropathology and motor coordination impairments, through the enhancement of neurotrophic factors levels and reduction of neuroinflammation^5^. To further demonstrate the relevance for human disease, in the present work, human induced pluripotent stem cell (iPSC)-derived neuroepithelial stem cells (NESC) were established as a source of cells potentially free of some limitations associated with the fetal and embryonic human stem cells, namely ethical problems^6^. Somatic cells can be reprogrammed into iPSC by the forced expression of the 4 reprogramming factors Sox-2, Klf-4, Oct-4, and C-Myc^11^. Then, iPSC can be induced into NESC, cells of the neuroectodermal lineage^12^.

Human iPSC-derived cells are very promising cell sources for cell therapy. Nevertheless, there is a need to perform long-term studies assessing their therapeutic potency and safety in the cerebellum. Moreover, given the high costs of preclinical development, there is also the need to identify cell therapeutic potency predictive markers that might indicate from the established human cell lines which present higher therapeutic potential. Such markers would reduce the number of cell lines to be tested in vivo, decreasing the costs of preclinical development^13,14^.

Two major concerns in the preclinical development of cell-based medicinal products are safety and therapeutic potency estimation^7,14–16^. Regarding safety, the risk of tumor induction and immune activation are important aspects to have into account in the preclinical development of these medicines. Neural progenitors and neuroepithelial stem cells trigger their therapeutic effects through several mechanisms, such as by promoting the replacement of dead cells, and by bystander effects, such as enhancing neurotrophic factors, reducing neuroinflammation, and increasing the neurogenesis. Thus, the therapeutic potency estimation in cell therapy, comprising the intended cell biological effect and clinical response, can be challenging^7,14–16^.

Accordingly, the present work was focused on the long-term evaluation of human Control (CNT) and MJD iPSC-derived NESC for their potential to be used in the treatment of cerebellar diseases. Our data demonstrated, for the first time, that human iPSC-derived NESC are capable to sustain the therapeutic mechanisms for six months after transplantation in the adult mice’s cerebella. Namely, graft-derived neurons and a significant enhancement of the levels of neurotrophic factors, neurogenesis-related proteins, and anti-inflammatory interleukins was found in the mice cerebella six months after cell transplantation.

## Results

### Human CNT and MJD iPSC-derived NESC phenotype and chromosomal stability evaluation

**Figure 1:**
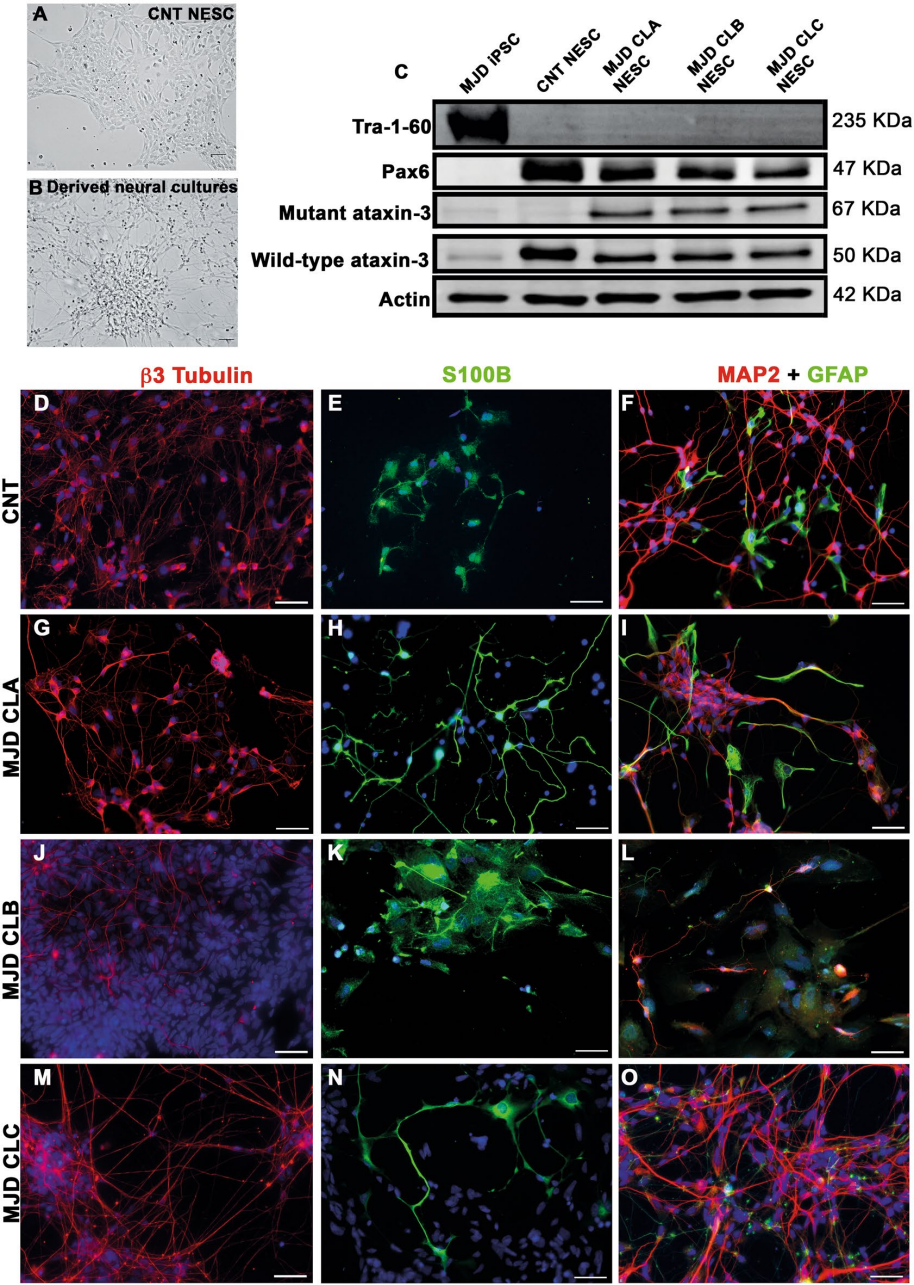
Human iPSC-derived NESC are multipotent and differentiate into neurons and glia. Control (CNT NESC, CNT) and MJD (MJD CLA, MJD CLB, and MJD CLC NESC) iPSC-derived NESC were established from Control and MJD fibroblasts, respectively, through cell reprogramming. Representative widefield microscopy image of (**A**) CNT NESC and their (**B**) derived neural cultures after 3 weeks of differentiation. (**C**) Representative image of western blot analysis showing no pluripotency marker Tra-1–60 in NESC, while the multipotency neuroectodermal marker Pax6 is present in these cells. Data also show the expression of mutant ataxin-3 and wild-type ataxin-3 in the MJD NESC (n = 3 independent experiments). (**D-O**) Immunofluorescence images of CNT and MJD NESC upon 3 weeks of differentiation exhibiting neurons shown by β3 Tubulin and MAP2 (red) and glia shown by S100B and GFAP (green). DAPI: blue, representative images of 3 independent experiments, scale bars: 50 μm.

Four human iPSC-derived NES cell lines, one Control (CNT NESC) and three MJD (MJD CLA, MJD CLB, and MJD CLC NESC), were established. These cells exhibit the characteristic morphology of NESC^12^ growing in monolayer (Fig. 1A), are capable of self-renewal and expansion, and differentiate into neural cultures (Fig. 1B). Western blot analysis demonstrated that CNT and MJD NESC have the multipotency neuroectodermal marker Pax6 and do not exhibit the pluripotency marker Tra-1–60 (Fig. 1C). As expected, the mutant ataxin-3 protein is present in the three MJD cell lines and is absent in the CNT NESC. No significant differences were detected for mutant Ataxin-3, Wild-type Ataxin-3, and Pax6 protein levels between the different cell lines (Supplementary Fig. S1). Control (Fig. 1D–F) and MJD (Fig. 1G–O) NESC differentiated into neurons, positive for MAP2 and β3 Tubulin, and into S100B- and GFAP-positive glia. While all cell lines originated astrocytes, as indicated by the S100B marker presence, the oligodendrocyte progenitors marker O4 was only detected in a few cells of the MJD CLB NESC differentiated cultures (Supplementary Fig. S2). These results demonstrated that the Control and MJD NESC are multipotent stem cells that upon in vitro differentiation originate neurons and astrocytes.

The four cell lines were evaluated for chromosomal stability (Supplementary Fig. S2). For the CNT, MJD CLB, and MJD CLC NESC a normal chromosomal composition was observed. The MJD CLA NESC exhibited a single clone with an abnormal chromosomal constitution. For the latter, a chromosome 14 derived from a reciprocal and unbalanced translocation between the long arms of chromosome 1 and chromosome 14, with probable breakpoints at 1q12 and 14q13, was detected. This modification resulted in a partial trisomy of the long arm of chromosome 1 and partial monosomy of the long arm of chromosome 14. The MJD CLA cell line was included in the study to investigate whether this modification would result in impairments in the functionality of the cells.

### Characterization of the human CNT and MJD neurons obtained from iPSC-derived NESC

**Figure 2:**
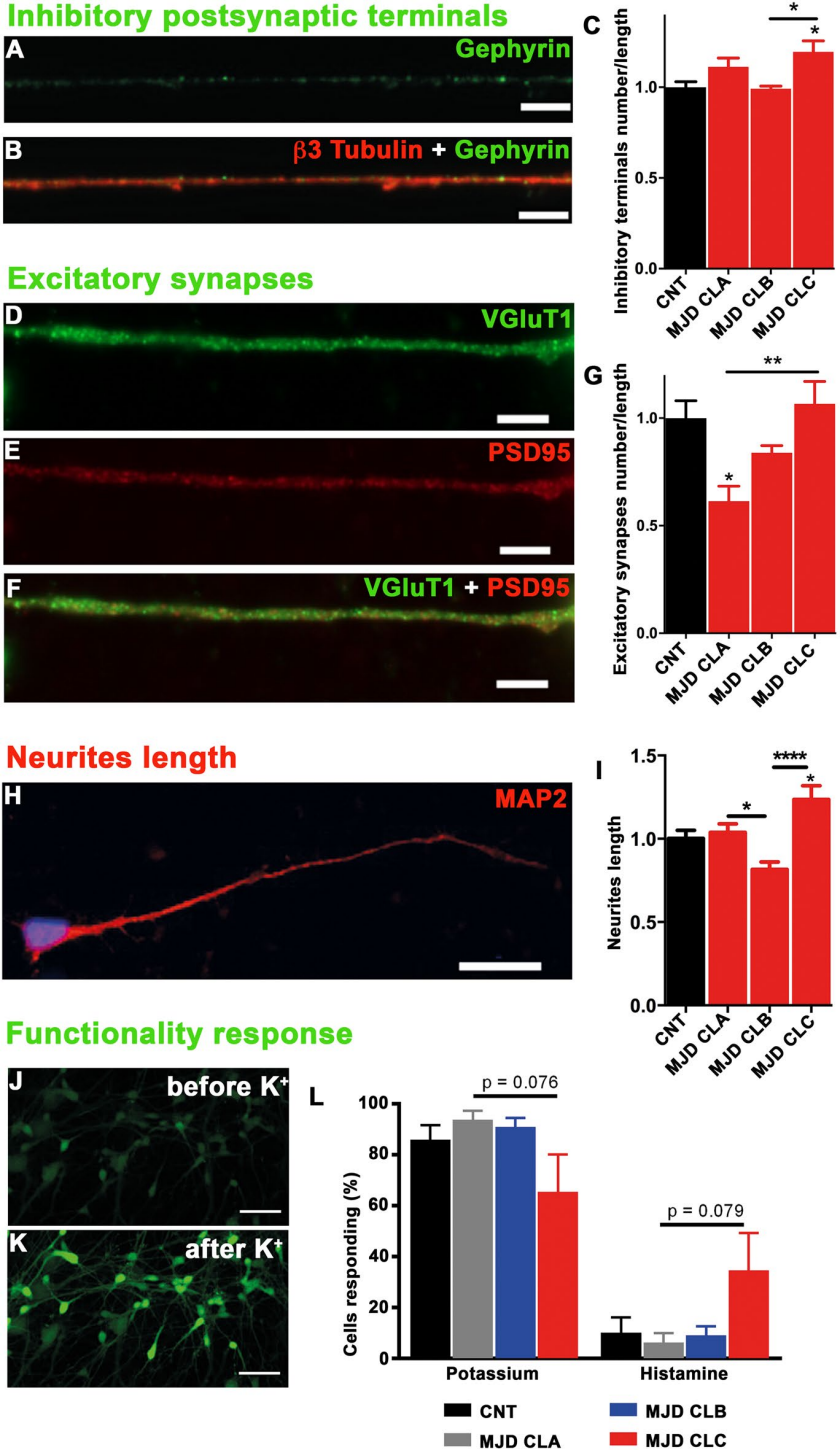
Evaluation of synapses, neurite length, and neuronal firing in Control and MJD neurons. Control, MJD CLA, MJD CLB, and MJD CLC iPSC-derived NESC (CNT, MJD CLA, MJD CLB, and MJD CLC, respectively) were differentiated into neural cultures for 3 weeks. Representative immunofluorescence images showing (**A**) inhibitory postsynaptic terminals, defined as gephyrin-positive (green) puncta on (**B**, merge) β3 Tubulin-positive (red) neurite. (**C**) Number of inhibitory postsynaptic terminals per neurite length normalized for CNT NESC (n = 3–4 independent experiments, analyzed neurons: CNT n = 40, MJD CLA n = 22, MJD CLB n = 41, MJD CLC n = 22). Representative immunofluorescence images of excitatory synapses, defined as instances of (**D**) VGluT1 (green) and (**E**) PSD95 (red) puncta (**F**) colocalization on MAP2-positive neurite. (**G**) Number of excitatory synapses per neurite length normalized for CNT NESC (n = 3–4 independent experiments, analyzed neurons: CNT n = 23, NESC CLA n = 21, MJD CLB n = 33, MJD CLC n = 29). (**A**,** B** and **D-F**) scale bars: 10 μm. (**H**) Representative immunofluorescence image of MAP2-positive (red) neurite of CNT NESC. (**I**) Total neurites length per neuron normalized for CNT NESC (n = 2–5 independent experiments, analyzed neurites: CNT n = 128, MJD CLA n = 197, MJD CLB n = 107, MJD CLC n = 101). Scale bar: 20 μm, DAPI: blue. (**J-L**) Neuronal firing evaluation through the variation of intracellular calcium concentration in neurons by single-cell calcium imaging. Representative images of the calcium fluorescence indicator (Fluo-4, green) in neurons (**J**) before and (**K**) after potassium (K^+^) stimulus. (**L**) Percentage of cells responding to potassium (neurons) and histamine (neural progenitors) stimulus (n = 4 independent experiments, analyzed neurons: CNT n = 271, MJD CLA n = 271, MJD CLB n = 59, MJD CLC n = 138); (**J**, **K**) scale bars: 50 μm. Data are expressed as mean ± SEM, *p < 0.05, **p < 0.01, and ****p < 0.0001, One-way ANOVA followed by Tukey’s post-test.

The ability to originate functional neurons upon brain transplantation is essential for cell-based therapies aiming at neuronal replacement. After 3 weeks of cell differentiation, the obtained neurons from Control and MJD NESC were evaluated for the neurites length and the number of inhibitory postsynaptic terminals, excitatory synapses, and functional neurons (Fig. 2).

Inhibitory postsynaptic terminals were detected in neurons obtained from the four cell lines (Fig. 2A–C). Significant differences in the number of inhibitory postsynaptic terminals per neurite length were observed between the cell lines, which did not directly correlate with the presence of mutant ataxin-3, given that the MJD CLC neurons exhibited a 1.20 times higher number of inhibitory postsynaptic terminals compared to MJD CLB neurons (Fig. 2C). Excitatory synapses were also detected in neurons of the four cell lines (Fig. 2D–G). MJD CLA neurons presented 38.62% fewer excitatory synapses as compared to CNT neurons and 45.29% fewer excitatory synapses than MJD CLC neurons (Fig. 2G). Differences in the neurites length of neurons obtained from the different cell lines were also detected (Fig. 2H and I). MJD CLC neurons presented 1.24-fold longer neurites than CNT neurons. While, MJD CLB neurons presented 22.28% and 42.10% smaller neurites as compared to MJD CLA and MJD CLC neurons, respectively (Fig. 2I).

Regarding the neuronal functionality response, single-cell calcium imaging was used to investigate the presence of neurons firing upon stimulation with potassium (50 mM KCl), an indication of functional neurons. While cell response to histamine indicate that cells remain undifferentiated as neural progenitors^5,17^ (Fig. 2J–L). All cell lines originated functional neurons and no significant differences in the number of cells responding as functional neurons or as neural progenitor cells were detected between the cell lines (Fig. 2L).

Inhibitory postsynaptic terminals, excitatory synapses, and firing neurons were also detected in neuronal cultures with 2 months (Supplementary Fig. S3) indicating the ability of human iPSC-derived NESC to originate functional neurons at this longer time point. Supplementary Table S1 summarizes the in vitro characterization of the cell lines.

### Cell phenotype and cell therapeutic potential evaluation in human brain organoids

**Figure 3:**
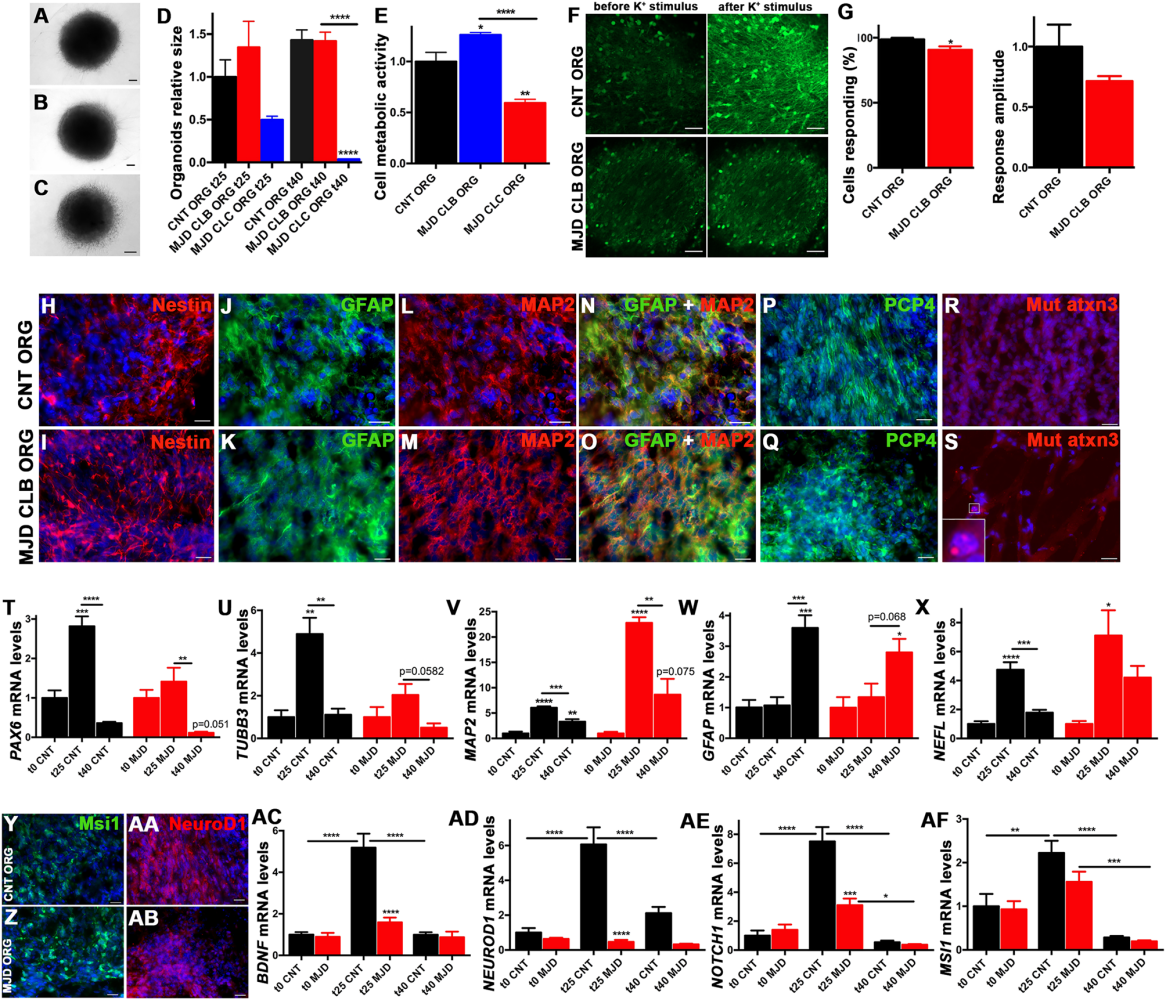
Evaluation of size, metabolic activity, neuronal firing, and expression of neurotrophic factors and neural progenitors proliferation markers in brain organoids established from human iPSC-derived NESC**.** Human brain organoids were established from the Control, MJD CLB, and MJD CLC iPSC-derived NESC (CNT ORG, MJD CLB ORG, and MJD CLC ORG, respectively) and cultured for 25 (t25) and 40 (t40) days. Representative widefield pictures of (**A**) CNT, (**B)** MJD CLB, and (**C**) MJD CLC organoids with 25 days, scale bars: 100 μm (n = 18–36 organoids/condition from 3 independent experiments). (**D**) Organoids’ area normalized for CNT ORG. (**E**) Cell metabolic activity evaluation through resazurin reduction assay, normalized for CNT ORG, n = 5 independent experiments. **(F**) Representative pictures showing the fluorescence calcium probe (Fluo-4, green) in single-cell calcium imaging analysis of CNT ORG and MJD CLB before and after potassium (K^+^) stimulus, showing organoids’ response to potassium at day 40 (CNT ORG n = 4, MJD CLB ORG n = 5 independent experiments). (**G**) Percentage of cells responding to potassium in CNT and MJD CLB ORG and response amplitude normalized for CNT ORG. Data showed a higher neuronal response in CNT ORG; n = 4–5/group. (**H–S**) Representative immunofluorescence images of CNT ORG and MJD CLB ORG with 40 days analyzed for (**H** and **I**) Nestin (red), (**J** and** K**) GFAP (green), (**L-O**) MAP2 (red), **(P** and **Q**) PCP4 (green), and (**R** and** S)** mutant ataxin-3 inclusions (red); lower insert in (**S**) is an image detail showing mutant ataxin-3 inclusions; n = 3 independent experiments. (**T-X**) and (**AC-AF**) mRNA levels evaluation of CNT and MJD CLB iPSC-derived NESC (t0) and organoids with 25 (t25) and 40 (t40) days by RT-qPCR. **T**) *PAX6*, **U**) *TUBB3,* **V**) *MAP2*, (**W**) *GFAP*, and **X**) *NEFL* mRNA levels normalized for t0 CNT (for CNT ORG) and t0 MJD (for MJD ORG); n = 4–5 independent experiments. (**Y-AB**) Representative immunofluorescence pictures showing (**Y and Z**) Msi1 (green) and (**AA** and **AB**) NeuroD1 (red) protein expression in CNT ORG and MJD CLB ORG; n = 3 independent experiments. (**AC**) *BDNF*, **(AD**) *NEUROD1*, (**AE**) *NOTCH1,* and (**AF**) *MSI1* mRNA levels, normalized for t0 CNT, showing higher mRNA levels in CNT ORG; n = 4–5 independent experiments. Scale bars: 50 μm, DAPI: blue; Data are expressed as mean ± SEM, *p < 0.05, **p < 0.01, ***p < 0.001, and ****p < 0.0001, (G) unpaired t-test with Welch's correction, and (D, E, T-X, and AC-AF) One-way ANOVA followed by Tukey’s post-test.

Brain organoids were established from the iPSC-derived NESC with the purpose to challenge the cell lines for their ability to originate neurons and trigger the bystander effects in a 3D experimental model closer to the brain than 2D cell cultures^18^. A protocol previously published to obtain brain organoids with human iPSC-derived NESC was used^19^. Thus, brain organoids derived from Control (CNT ORG), MJD CLB (MJD CLB ORG), and MJD CLC (MJD CLC ORG) iPSC-derived NESC were obtained (Fig. 3A–C) and characterized. Interestingly, at day 40, MJD CLC ORG were 38.65 times smaller than CNT ORG and 38.32 times smaller than MJD CLB ORG (Fig. 3D), suggesting a lower 3D proliferation and organization ability of MJD CLC NESC. Moreover, MJD CLC organoids exhibited a 40.70% lower cell metabolic activity at day 40 (Fig. 3E). The MJD CLB organoids presented a 26.20% higher metabolic activity as compared to CNT ORG, even though no significant differences in size were detected between MJD CLB and CNT ORG (Fig. 3D). Through single-cell calcium-imaging analysis (Fig. 3F and G), MJD CLB organoids exhibited 8.06% fewer firing neurons, as compared to Control organoids. While no significant difference was observed in the response amplitude to potassium between CNT and MJD CLB ORG (Fig. 3G).

Immunohistochemistry revealed that both Control and MJD CLB organoids exhibit Nestin-positive progenitor cells (Fig. 3H and I), GFAP-positive glia (Fig. 3J, K, N and O), and MAP2- and PCP4-positive neurons (Fig. 3L–Q). Regarding the presence of mutant ataxin-3 protein inclusions (Fig. 3R and S) in the MJD organoids, a few mutant ataxin-3 inclusions were observed. Moreover, the mRNA levels of CNT and MJD CLB ORG at different maturation stages (0, 25, and 40 days in culture) indicated a similar cellular specialization profile for both CNT and MJD CLB organoids. A significant reduction in the *PAX6* mRNA levels, a marker of neuroectodermal progenitor cells, was observed for CNT and MJD CLB ORG as organoids mature from 25 to 40 days (Fig. 3T). The neuronal markers *TUBB3* (Fig. 3U) and *MAP2* (Fig. 3V) mRNA levels exhibited a significant increase at day 25, while the glial marker *GFAP* mRNA (Fig. 3W) levels were only found significantly enhanced at day 40. Thus, the established organoids recapitulated the embryogenesis feature of neurons appearing before glial cells^20,21^. A similar profile was obtained for the NEFL mRNA levels in CNT and MJD CLB ORG, coding the neurofilament light chain protein associated with axonal damage and disease-related structural brain changes^22^ (Fig. 3X).

Subsequently, CNT and MJD CLB organoids were assessed for molecules enrolled in the bystander effects triggered by neural stem cells (Fig. 3Y-AF). Expression of the neural progenitors’ proliferation marker Musashi1 (Msi1; Fig. 3Y and Z) and neuronal differentiation marker NeuroD1 (Fig. 3AA and AB) was observed for both CNT and MJD CLB ORG at day 40. Regarding mRNA levels, at day 25 the CNT ORG showed 3.27 times higher Brain-derived neurotrophic factor (*BDNF*) levels (Fig. 3AC), as well as 12.99 and 2.42 times higher levels of *NEUROD1* (Fig. 3AD) and *NOTCH1* (also a neural progenitors’ proliferation marker) (Fig. 3AE), respectively, compared with MJD ORG. No significant differences were observed between CNT and MJD CLB ORG for the *MSI1* mRNA levels (Fig. 3AF).

These results indicate that both cell lines are capable to originate glia and neurons in brain organoids. Nevertheless, higher levels of neurotrophic factors and markers of neural progenitors’ proliferation and neuronal differentiation were detected in the organoids derived from CNT NESC, suggesting that this cell line has a higher potential to trigger these therapeutic mechanisms in a 3D brain model.

### Survival and safety of human CNT and MJD iPSC-derived NESC upon cerebellar transplantation

The four cell lines, CNT, MJD CLA, MJD CLB, and MJD CLC NESC, submitted to a maturation protocol before cerebellar transplantation, were characterized for MAP2 and GFAP mRNA and protein expression (Supplementary Fig. S4). Regarding *MAP2* and *GFAP* mRNA levels, a tendency for increased levels was observed for most cell lines, while MJD CLB NESC showed significant 5.53-fold higher *MAP2* mRNA levels upon maturation. Cell cultures showed a higher percentage of MAP2-positive neurons as compared to GFAP-positive astrocytes. Namely, MJD CLB and MJD CLC NESC showed 4.54- and 8.74-fold higher percentages of MAP2-positive cells as compared to GFAP-positive cells. MJD CLB and MJD CLC NESC also presented higher percentages of MAP2-positive cells compared to CNT NESC.

To evaluate the effect of cells in vivo, NESC (expressing GFP) were transplanted in 5–6-week-old NOD.SCID mice and the animals were studied at two and six months after cell grafting. At the 2-month time point, the following animals were transplanted CNT NESC (n = 4), MJD CLA NESC (n = 3), MJD CLB (n = 5), and MJD CLC NESC (n = 4). For the 6-month time point were transplanted CNT NESC (n = 5), MJD CLA NESC (n = 3), and HBSS-injected controls (n = 4).

**Figure 4:**
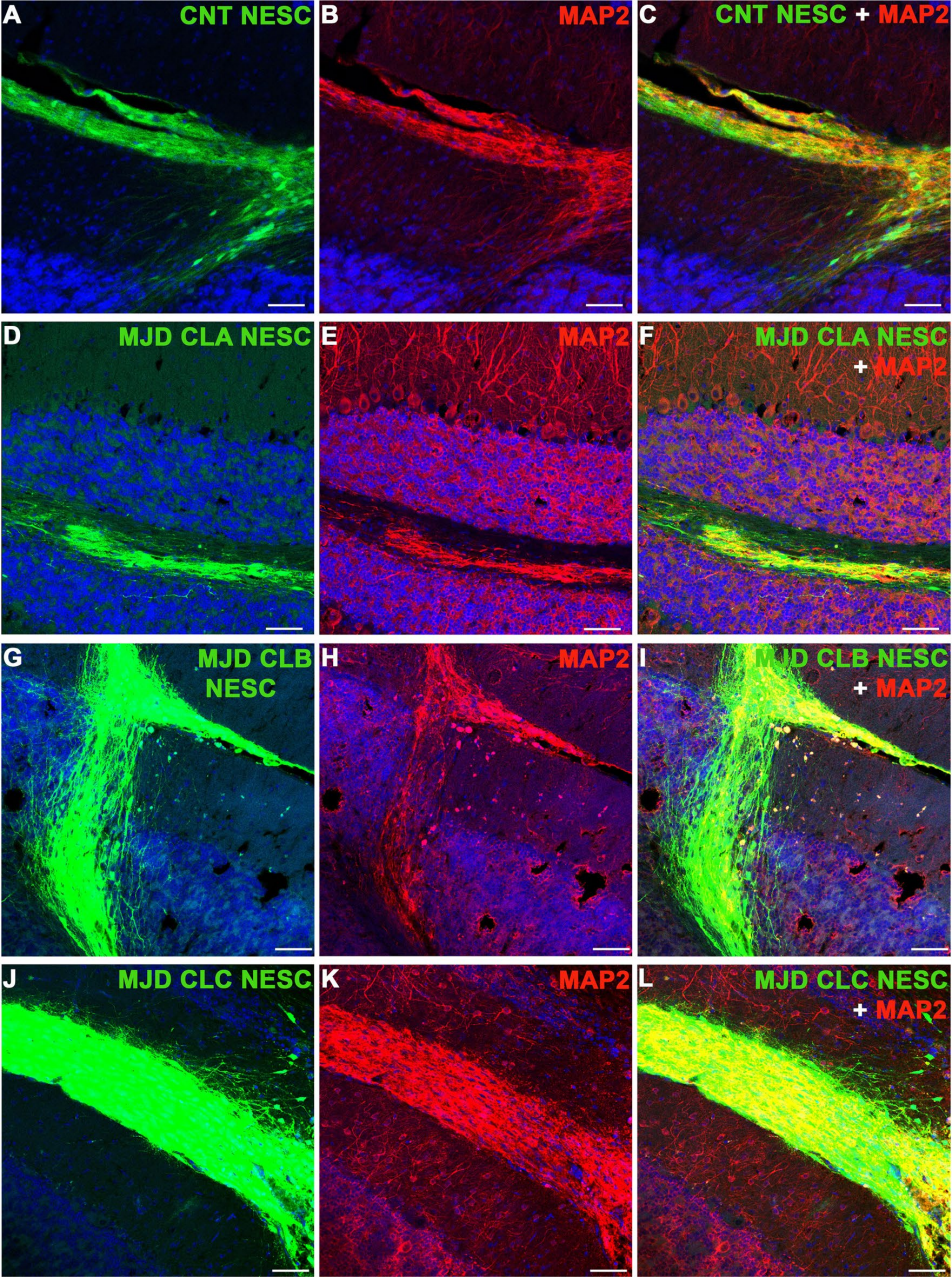
Neuronal differentiation of Control and MJD iPSC-derived NESC in the cerebellum of adult mice. Representative immunofluorescence confocal microscopy images of MAP2 labeling in mice cerebellar sections two months after cell transplantation. (**A-C**) Control (CNT NESC), (**D-F**) MJD CLA (MJD CLA NESC), (**G-I**) MJD CLB (MJD CLB NESC), and (**J-L**) MJD CLC (MJD CLC NESC) iPSC-derived NESC expressing GFP (green) survived for 2 months and differentiated into MAP2-positive (red) neurons showed by the colocalization between GFP and MAP2. DAPI: blue, n = 3 mice/group, Scale bars: 100 μm.

**Figure 5:**
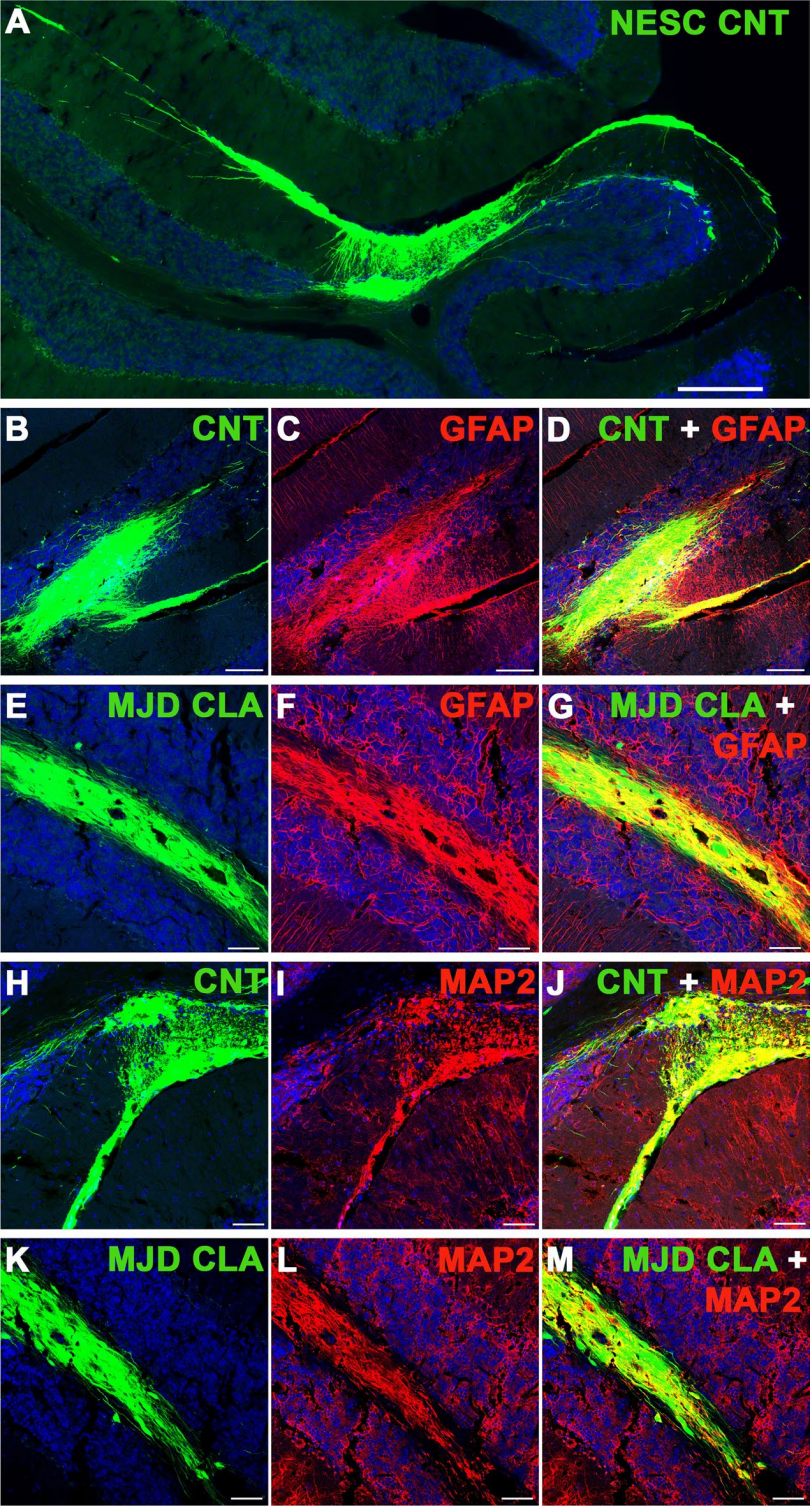
Human glia and neurons detected in mice cerebella six months after Control and MJD iPSC-derived NESC transplantation. (**A-D and H-J**) Control and (**E–G** and **K-M**) MJD CLA iPSC-derived NESC expressing GFP (green) were detected in the cerebellum six months after transplantation. (**A**) Representative fluorescence image illustrating the limited migration of the grafted cells (green) in the adult cerebellum. Representative immunofluorescence confocal microscopy images of (**B-G**) GFAP (red) and (**H-M**) MAP2 (red) labeling. Images show that six months after cell transplantation, Control (CNT) and MJD CLA iPSC-derived NESC grafts have GFAP-positive glial cells and MAP2-positive neurons showed by the colocalization between GFP and GFAP and MAP2, respectively. DAPI: blue, n = 3 mice/group, scale bars: (**A**) 200 μm, (**B-M**) 100 μm.

**Figure 6:**
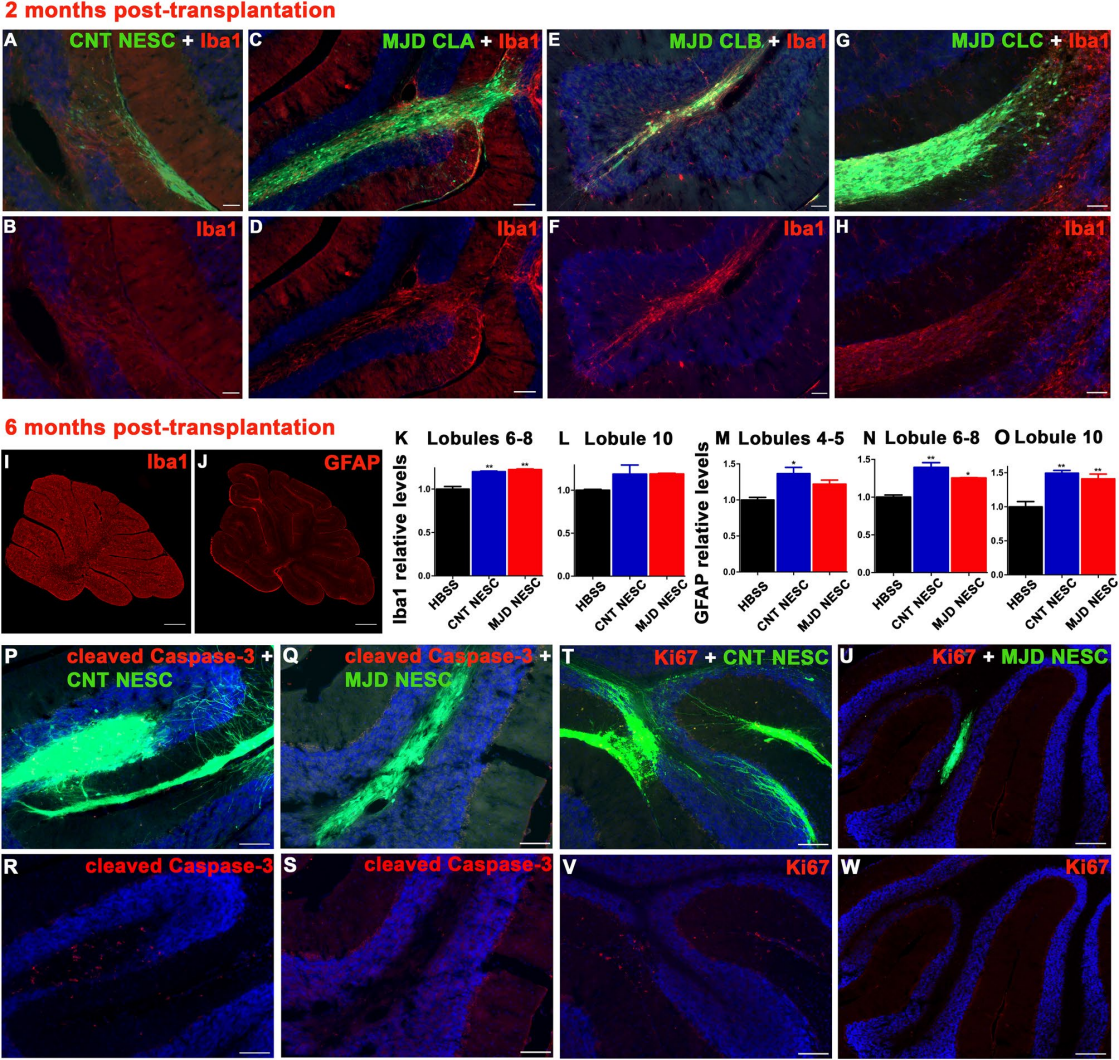
Evaluation of microglia recruitment, astrogliosis, cell overproliferation, and cell death in iPSC-derived NESC grafts. **(A-H**) Two and (**I-W**) six months after the cerebellar transplantation of Control and MJD iPSC-derived NESC expressing GFP (green), mice cerebellar sections were evaluated for safety parameters. (**A-H**) Representative fluorescence microscopy images of Iba1 (red) immunolabeling in mice cerebella transplanted with (**A** and **B**) CNT NESC, (**C** and** D**) MJD CLA NESC, (**E** and** F**) MJD CLB NESC, and (**G** and** H**) MJD CLC NESC, n = 3 mice/group. Representative fluorescence microscopy pictures of (**I**) Iba1 and (**J**) GFAP (red) immunolabeling in mice cerebella transplanted with CNT NESC. Mice transplanted with CNT NESC and MJD NESC, and control mice injected with HBSS, were evaluated for Iba1 levels in cerebellar lobules (**K**) 6–8 and (**L**) 10 and for GFAP levels in cerebellar lobules (**M**) 4–5, (**N**) 6–8, and (**O**) 10. (**K–O**) Iba1 and GFAP relative levels were quantified through cerebellar lobules medium fluorescence intensity normalized for the HBSS-injected NOD.SCID mice (HBSS); n = 3 mice/group. Data are expressed as mean ± SEM, *p < 0.05 and **p < 0.01, One-way ANOVA followed by Tukey’s post-test. Representative fluorescence confocal microscopy pictures of (**P-S**) cleaved Caspase-3 (red) and (**T-W**) Ki67 (red) immunolabeling in the cell grafts (green). Scale bars: 100 μm, n = 3 mice/group.

The four cell lines were tested for survival and ability to differentiate into glia and neurons at 2 months post-transplantation in the cerebellum. All cell lines survived and differentiated into MAP2-positive neurons (Fig. 4) and GFAP-positive glia (Supplementary Fig. S5) observed in healthy grafts presenting strong GFP expression and neuronal projections. Moreover, the evaluation of the number of graft-derived cells differentiated into neurons, given by colocalization between the human nuclei antigen (HuNu) and β3 Tubulin and NeuN, revealed an 85.03 – 90.2% and 76.08 – 97.17% (depending on the cell line) differentiation into neurons, respectively (Supplementary Fig. S6). The colocalization between HuNu and S100B showed a 15.56 – 31.67% differentiation into astrocytes. Finally, a small percentage (0 – 0.040%) of undifferentiated (Pax6-positive) human cells were detected for all cell lines. Afterward, a longer-term evaluation of CNT and MJD CLA NESC survival and differentiation was performed 6 months post-transplantation. Strong GFP expression and neuronal projections were also detected in the grafts. Cell migration was mostly limited to the area surrounding the transplantation site (lobule 5) (Fig. 5A). Human cells differentiated into glia (Fig. 5B–G) and neurons were detected (Fig. 5H–M), indicating that grafted NESC differentiated into human neurons and glia that survived for six months in mice cerebella. Regarding the potential neuroinflammation triggered by the cells, at 2 months post-transplantation some microglia (Iba1-positive cells) infiltration was detected in the grafts of CNT (Fig. 6A and B) and MJD CLA (Fig. 6C and D), CLB (Fig. 6E and F), and CLC (Fig. 6G and H) NESC. At 6 months post-transplantation, the cerebella of mice transplanted with CNT and MJD CLA NESC exhibited a 1.201 and 1.225 times microglia levels enhancement (on lobules 6–8), respectively, compared with HBSS-injected mice (Fig. 6I and K). Additionally, no significant Iba1 enhancement was detected in lobule 10 (Fig. 6L), the lobule more distant from the graft, indicating that the microglia recruitment is limited to the lobules near the graft zone. Moreover, CNT and MJD NESC-mediated augmentation of astrocyte (GFAP-positive cells) levels (Fig. 6J and M–O) was observed at lobules 4–5, 6–8, and 10.

Cell death assessment provides a direct indication of immune rejection and viability of the transplanted cells. At 6 months post-transplantation the majority of the observed graft-derived cells were alive, since GFP-positive cells exhibited minor colocalization with the apoptosis marker cleaved Caspase-3 (Fig. 6P-S). Regarding graft overproliferation, indicated by the presence of proliferating (Ki67-positive) cells in the grafts, it was detected some proliferating cells, namely in the CNT NESC-derived grafts (Fig. 6T and V). Nevertheless, no Ki67-positive mass of cells was observed that would indicate the presence of tumors (Fig. 6T–W).

### Differentiation of CNT and MJD iPSC-derived NESC in cerebellar neurons and establishment of synapses in the host cerebella

**Figure 7:**
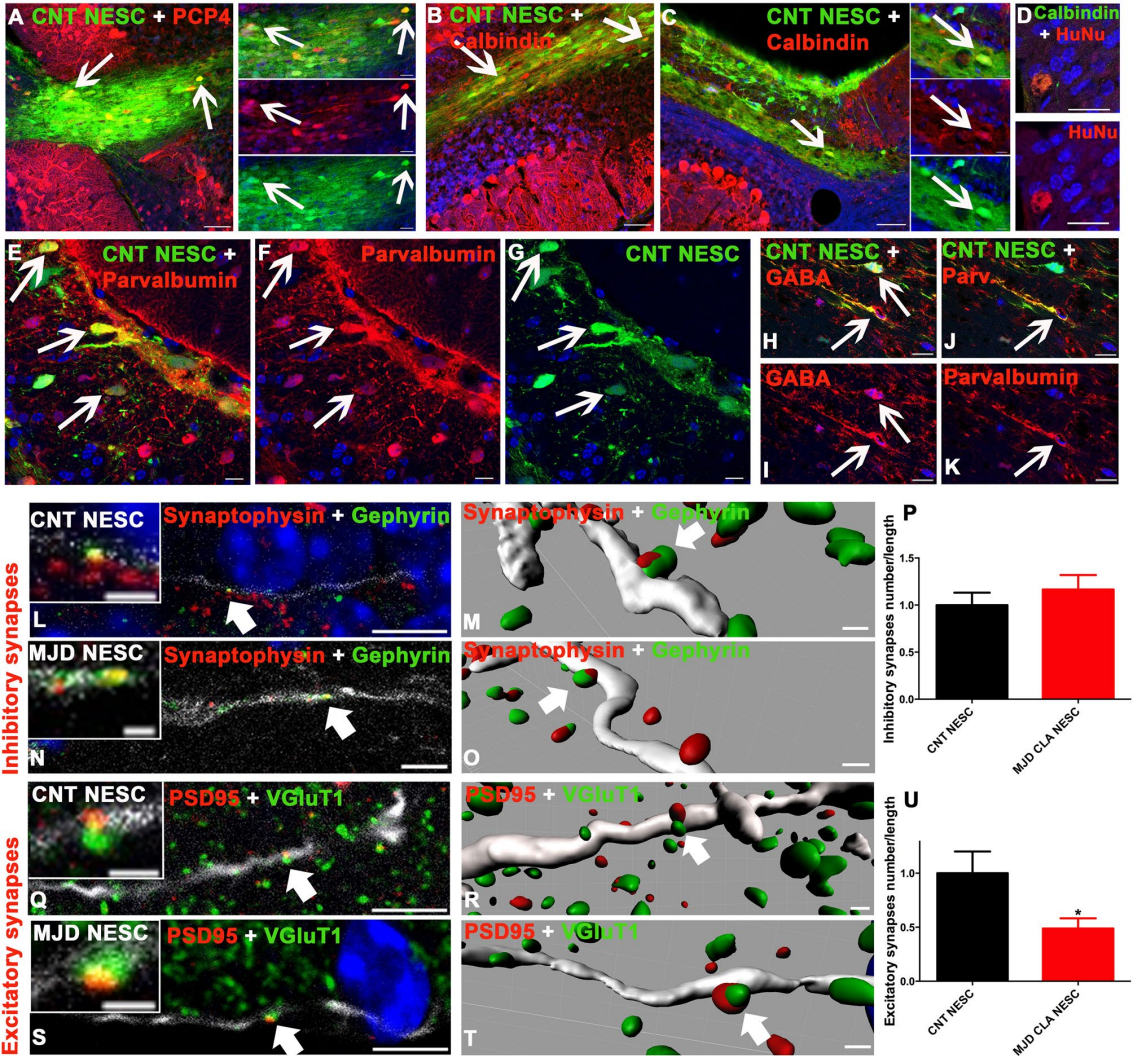
Human iPSC-derived NESC differentiate into neurons expressing Purkinje cell markers and the new neurons establish inhibitory and excitatory synapses in mice cerebella. CNT (CNT NESC) and MJD CLA (MJD NESC) iPSC-derived NESC expressing GFP (green) transplantation, mice cerebella were evaluated for cerebellar neurons differentiation and synapses establishment. Representative immunofluorescence confocal microscopy images showing that the grafted cells differentiated into (**A**) PCP4- and (**B-D**) Calbindin-positive (red) cells, markers of Purkinje cells, and in (**E–G, J and K**) Parvalbumin- and (**H and I**) GABA-positive (red) cells, showed by the colocalization between GFP (green) and the cell markers (red). D) Representative immunofluorescence confocal microscopy image of human nuclei antigen (HuNu) colocalization with Calbindin. Cerebellar sections analyzed **A, C-U**) six and **B**) two months after cell transplantation. Scale bars: (**A-C**) 50 μm, (**D**) 20 μm, and **(E-K**) 10 μm. The lateral panels in (**A**) and (C) exhibit individual color channels of the cells indicated by the white arrows in the graft, scale bars: 20 μm. White arrows show cells positive for the cell markers. (**L**, **N**, **Q,** and **S**) Representative immunofluorescence confocal microscopy images showing (**L** and **N**) colocalization of puncta positive for presynaptic Synaptophysin (red) and postsynaptic Gephyrin (green) displaying inhibitory synapses in neurites (GFP, white) derived from (**L**) CNT NESC and (**N**) MJD NESC transplanted in cerebella. Scale bars: 5 μm, upper small inserts: 1 μm. **M** and **O**) 3D representations of Z-stack confocal images, using Imaris software, of presynaptic Synaptophysin (red) and postsynaptic Gephyrin (green) immunolabeling showing inhibitory synapses in neurites (GFP, white) derived from CNT and MJD NESC grafted cells, respectively. Scale bars: (**M**) 2 μm, **O**) 0.5 μm; the upper small inserts show a higher magnification of the synapses indicated by the white arrows. (**P**) Inhibitory synapses number per neurite length normalized for CNT NESC; CNT NESC n = 22 neurites from 3 grafted mice, MJD CLA NESC n = 14 neurites from 3 grafted mice. (**Q and S**) Colocalization of puncta positive for presynaptic VGluT1 (green) and postsynaptic PSD95 (red) showing excitatory synapses in neurites (GFP, white) derived from (**Q**) CNT NESC and (**S**) MJD NESC transplanted in cerebella. Scale bars: 5 μm, upper small inserts: 1 μm; the upper small inserts show a higher magnification of the synapses indicated by the white arrows. (**R** and** T**) 3D representations of Z-stack confocal images, using Imaris software, of presynaptic VGluT1 (green) and postsynaptic PSD95 (red) immunolabeling showing excitatory synapses in neurites (GFP, white) derived from CNT and MJD NESC grafted cells, respectively; scale bars: 0.5 μm. **U**) Excitatory synapses number per neurite length normalized for CNT NESC; CNT NESC n = 17 neurites from 3 grafted mice, MJD CLA NESC n = 14 neurites from 3 grafted mice. DAPI: blue, Data are expressed as mean ± SEM, *p < 0.05, Unpaired t-test with Welch's correction.

Purkinje cells are the neurons responsible for the integration of the cerebellar afferent and efferent neuronal pathways^23^. GABA-positive inhibitory cerebellar neurons include stellate cells, basket cells, Purkinje cells, and neurons in the deep cerebellar nuclei. Parvalbumin is present in some GABAergic interneurons, such as the basket cells and stellate cells located at the cerebellar molecular layer^24^. Six months after iPSC-derived NESC transplantation, sections of mice cerebella exhibited graft-derived cells (expressing GFP) positive for markers of cerebellar neurons, such as Purkinje cells (PCP4- and Calbindin-positive cells) (Fig. 7A, C-D), and interneurons (Parvalbumin- (Fig. 7E–G, J and K) and GABA- (Fig. 7H and I) positive neurons). Moreover, Calbindin-positive cells were also detected at earlier time points (2 months post-transplantation) (Fig. 7B) and some graft-derived cells were detected in the Purkinje cell layer (Figure S5). No binucleated cells labeled for human nuclei (HuNu) and Calbindin were observed (Fig. 7D), indicating that these cells are not derived from the fusion between human NESC and mice neurons or the transfer of GFP protein from the human NESC to cerebellar neurons of mice. The ability of the graft-derived neurons to establish inhibitory (Fig. 7L–P) and excitatory (Fig. 7Q–U) synapses in mice’s cerebella was also evaluated. Colocalization between Synaptophysin-positive presynaptic puncta and Gephyrin-positive postsynaptic terminals was found in GFP-expressing neurites (white, graft), showing the formation of inhibitory synapses in the human graft-derived neurons in mice cerebella. The synapses' 3D representation and quantification revealed no significant difference in the number of inhibitory synapses between CNT and MJD NESC-derived neurons (Fig. 7P). Additionally, colocalization between PSD95-positive postsynaptic puncta and VGluT1-positive presynaptic terminals was found in GFP-expressing neurites, showing the formation of excitatory synapses in the human graft-derived neurons in mice cerebella. The respective quantification revealed that MJD NESC-derived neurons present 51.2% fewer excitatory synapses compared with CNT NESC (Fig. 7U). Interestingly, these results correlate with the in vitro data showing that MJD CLA NESC-derived neurons present a lower number of excitatory synapses (Fig. 2G).

### Modulation of neurotrophic factors, inflammatory interleukins, and neurogenesis in the host cerebella promoted by human iPSC-derived NESC grafts

**Figure 8:**
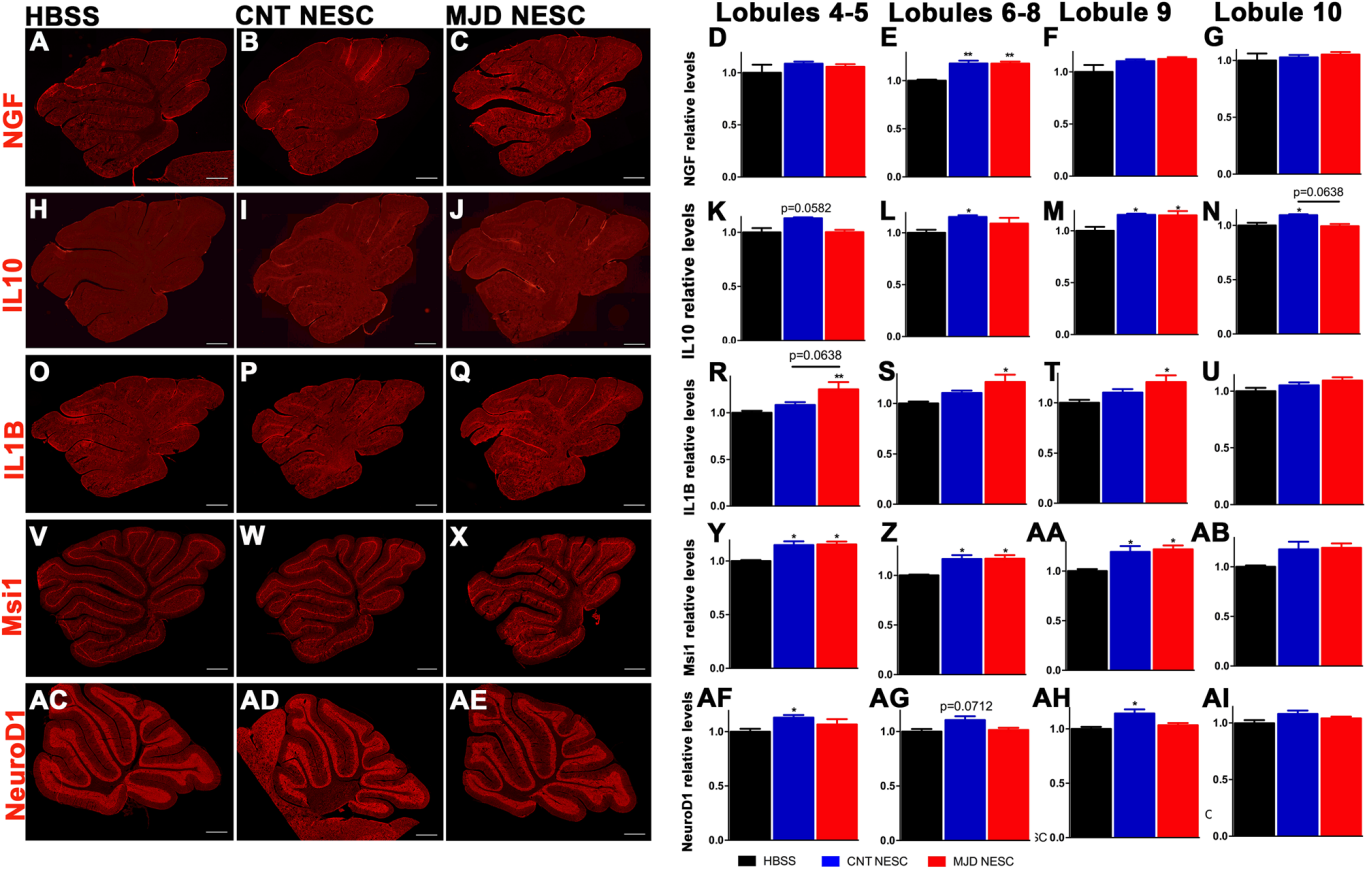
Human iPSC-derived NESC grafts maintained the bystander effects in mice cerebella for six months. (**A-AI**) Six months after Control (CNT NESC) and MJD CLA (MJD NESC) iPSC-derived NESC transplantation in the cerebellum of adult NOD.SCID mice, the cerebellar levels of the neurotrophic factor NGF, anti- (IL10) and pro- (IL1B) inflammatory interleukins, and neurogenesis-related proteins (Msi1 and NeuroD1) were measured. Representative immunofluorescence microscopy images of (**A**-**C**) NGF, (**H-J**) IL10, (**O-Q**) IL1B, (**V-X**) Msi1, and (**AC-AE**) NeuroD1 labeling in the cerebellum of mice injected with (**A**, **H**, **O**, **V,** and **AC**) HBSS and transplanted with (**B**, **I**, **P**, **W**, and **AD**) CNT NESC and (**C**, **J**, **Q**, **X**, **AE**) MJD NESC. Scale bars: 500 μm. Levels of (**D-G**) NGF, (**K-N**) IL10, (**R-U**) IL1B, (**Y-AB**) Msi1, and (**AF-AI**) NeuroD1 in cerebellar lobules 4 and 5 (Lobules 4–5), 6 to 8 (Lobules 6–8), 9 (Lobule 9), and 10 (Lobule 10) measured through cerebellar lobules medium fluorescence intensity normalized for the HBSS group. Data revealed increased NGF, IL10, Msi1, and NeuroD1 levels in mice cerebella transplanted with CNT NESC and increased NGF, IL1B, and Msi1 levels in mice cerebella transplanted with MJD NESC; n = 3–5 mice/group. Data are expressed as mean ± SEM, *p < 0.05 and **p < 0.01, One-way ANOVA followed by Tukey’s post-test.

The enhancement of neurotrophic factor levels is one of the therapeutic mechanisms expected from NESC transplantation. Data indicate that nerve growth factor (NGF) levels were 1.179 and 1.178 times increased by CNT NESC and MJD NESC grafts, respectively, in the lobules 6–8 (Fig. 8A–G). On the other hand, CNT NESC and MJD NESC mediated no significant changes in the levels of glial cell-derived neurotrophic factor (GDNF) (Supplementary Fig. S7).

Given the reported cross-talk communication between the immune system and neural stem cells^10^, the cerebellar levels of three interleukins markedly enrolled in neuroinflammation, IL10, IL1B, and IL6, were assessed. The cerebellar levels of the anti-inflammatory interleukin IL10 (Fig. 8H–N) were found increased in all lobules evaluated of mice transplanted with CNT NESC. Data demonstrated a 1.130, 1.153, 1.154, and 1.095 times increase of IL10 levels in lobules 4–5, 6–8, 9, and 10, respectively. Whereas, MJD NESC transplantation triggered IL10 levels enhancement only in lobule 9. CNT NESC promoted no significant changes in the levels of the pro-inflammatory interleukins IL1B (Fig. 8O–U) and IL6 (Supplementary Fig. S7); whereas the MJD NESC increased IL1B by 1.253, 1.214, and 1.208 times in lobules 4–5, 6–8, and 9, respectively, demonstrating that MJD NESC grafts increased neuroinflammation.

Finally, as stem cell transplantation activates neurogenesis, the cerebellar levels of Msi1 (Fig. 8V-AB), NeuroD1 (Fig. 8AC-AI), and Ki67 (Supplementary Fig. S7) were also evaluated in the cell-grafted mice. Both CNT and MJD NESC grafts significantly increased Msi1 cerebellar levels. Interestingly, the NeuroD1 levels were found 1.129 and 1.142 times enhanced in lobules 4–5 and 9, respectively, in the cerebellum of mice transplanted with CNT NESC. Whereas, no significant changes in the NeuroD1 cerebellar levels were promoted by MJD NESC transplantation. Regarding the cell proliferation marker Ki67, the mice transplanted with CNT NESC exhibited 1.127, 1.134, and 1.170 times higher Ki67 levels in lobules 4–5, 6–8, and 9, respectively, as compared with HBSS-injected mice. While the cerebellum of mice grafted with MJD NESC exhibited no significant changes in the Ki67 levels.

Altogether, these results indicate that CNT iPSC-derived NESC triggered a more robust enhancement of the stem cell bystander effects.

Regarding potential structural alterations triggered by cell implantation in the cerebellum, the cerebellar total area and the molecular and granular layers area in lobules 4 and 5 (cell graft zone) and lobules 9 (far from graft zone) were assessed (Supplementary Fig. S8). No significant differences were observed for both the cellular layers area and the cerebellar total area between the control mice (HBSS) and mice injected with CNT and MJD CLA NESC at six months post-transplantation.

Subsequently we looked into the iPSC-derived NESC gene expression of genes enrolled in neurogenesis, namely cell proliferation and neuronal maturation, to evaluated a potential correlation between the gene expression and the therapeutic potential. Thus, the *EN2, GBX2*, *MSI1*, *NOTCH1*, and *NEUROD1* mRNA levels were evaluated in CNT, MJD CLA (CLA), and MJD CLB (CLB) iPSC-derived NESC and in the respective differentiated cell cultures (Supplementary Fig. S9). In differentiated neural cultures, MJD CLA and MJD CLB NESC presented 5.43 and 7.19 times lower *EN2* mRNA levels, respectively, as compared to CNT NESC. No significant differences between the cell lines were observed for *NEUROD1* mRNA levels. Finally, in CLA NESC differentiated cells the *MSI1* and *NOTCH1* mRNA levels were 1.66 and 2.32 times lower, respectively (Supplementary Fig. S9). These results indicate that the MJD CLA iPSC-derived NESC, which demonstrated a lower ability to trigger the bystander effects in vivo, also exhibited lower mRNA expression of *EN2*, *MSI1*, and *NOTCH1*. Accordingly, this data suggests that the expression of these genes might be explored to predict the cell therapeutic potency of the established cell lines.

## Discussion

The lack of long-term studies assessing the therapeutic potency and safety of human cell sources after cerebellar transplantation impairs the application of cell therapy to cerebellar disease treatment. In the present work, we showed for the first time that human iPSC-derived NESC survive, differentiate into neurons expressing Purkinje cell markers as Calbindin and PCP4, and maintain bystander effects such as modulation of inflammatory interleukins, neurotrophic factors, and neurogenesis-related proteins, for up to 6 months post-transplantation in the cerebellum. Moreover, no signs of graft rejection or overgrowth were detected.

Cell reprogramming of somatic cells into undifferentiated cells, namely induced pluripotent stem cells (iPSC)^11,25^, represented a major breakthrough for cell-based therapies by providing new cell sources to be employed in the development of cell-based medicinal products (CBMP). Additionally, the establishment of patient-specific cells to be used for autologous transplantation can mitigate immunorejection problems observed in other cell sources^6,7^. CBMP heterogeneity in terms of their composition and therapeutic mechanisms renders their development more complex compared with classical medicinal compounds^26^.

In the present work, four human iPSC-derived neuroepithelial stem cell lines were established and characterized in vitro and in vivo for some of the critical parameters recognized by the CBMP development guidelines^7,14–16^, such as cell identity, therapeutic potency, and safety.

The multipotent neuroectodermal identity of the cell lines, CNT, MJD CLA, MJD CLB and MJD CLC iPSC-derived NESC, was confirmed by phenotypic characterization. The four cell lines are capable of self-renewal, express markers of multipotent neuroectodermal cells, and differentiate into glia and neurons. As expected, no oligodendrocytes were consistently detected in the cell cultures, since the establishment of oligodendrocytes from human iPSC-derived stem cells requires specific differentiation protocols^27,28^. The chromosomal stability of iPSC-derived cell lines is an important concern, given that genetic instability might result in tumorigenicity upon cell transplantation^13,29^. The MJD CLA cell line was found to have a reciprocal and unbalanced translocation between the long arms of chromosome 1 and chromosome 14. The specific impact of this modification on the therapeutic potency and safety is unknown to us, and difficult to predict even with the genome sequencing normally used to detect disease-causing mutations^30^.

Neurons obtained from the differentiation of the different cell lines exhibited differences in the number of synapses, neurite length, and functional neurons. Phenotype variation in human iPSC and their derived cells have been reported. The genetic identity of the cell donor impacts the phenotype of the derived cells (inter-patient variation). Nevertheless, variables such as insertional effects of the reprogramming factors resulting in variable epigenetic profiles can also result in cell phenotype heterogeneity even in cell lines obtained from the same patient (intra-patient variation)^31–33^. The latter can explain the differences observed between the MJD cell lines. Regarding the differences detected between CNT and MJD cell lines, besides the impact of the inter-patient variation in the cell phenotype, it is important to also have into consideration the reported synaptic impairments associated with the mutant ataxin-3 protein^34^. Nevertheless, since only one CNT cell line was used in this study, further experiments with more control cell lines are required to evaluate the mutant ataxin-3 impact in neurons obtained from iPSC-derived NESC.

Brain organoids provide important insights into the 3D organization and specialization of cells^21^. MJD CLB brain organoids presented no significant difference in size compared with CNT organoids, nonetheless exhibited higher metabolic activity measured by the resazurin reduction into resoflurin. Neurons and astrocytes have different metabolic pathway preferences^35^, which might translate into differences in the levels of metabolic intermediates produced, such as NADPH, NADH, and glutamate^35,36^ known to reduce resazurin. Thus, further experiments will be required to elucidate whether the difference observed in the organoids’ cell metabolic activity might be explained by a different prevalence of neurons and astrocytes.

The presence of protein inclusions and neuronal death is frequently associated with late stages of neurodegenerative diseases such as MJD^37–39^. This may explain the few mutant ataxin-3 inclusions detected in the MJD organoids, given that the organoids established from the MJD iPSC-derived NESC might require a longer maturation to present a stronger MJD phenotype.

Control and MJD iPSC-derived NESC clearly survive and differentiate into neurons and glia after cerebellar transplantation into adult immunocompromised NOD.SCID mice. Moreover, a small percentage of undifferentiated cells and a higher number of neurons as compared to astrocytes were observed in the human grafts. As we had a limited amount of cerebellar sections with grafts, it was not possible to compare the ability of all the different cell lines to differentiate into neurons and astrocytes. The observed high percentages of differentiated cells and low percentages of Pax6-positive cells, and since Pax6 plays an important role in cell migration^40,41^, suggest that at 2 months post-transplantation there is no significant migration observed among the grafted cells.

Regarding the neuroinflammation triggered by the grafts, there is always some degree of immune reaction against the human cells^42^. Moreover, astroglia recruitment around the grafts is not strictly harmful because astroglia plays a crucial role in the synaptic integration of the grafted cells and activates neurotrophic and anti-inflammatory and anti**‐**oxidant mechanisms^42^.

The presence of Ki67-positive cells in the grafts reveals that cells retained some proliferation capacity, even after brain transplantation. Increasing the maturation period of the cells before transplantation might reduce this proliferation capacity. Nevertheless, the enhancement of the cells’ maturation may result in the reduction of the transplantation success, as observed in other studies^43,44^.

Six months after iPSC-derived NESC transplantation, PCP4- and Calbindin-positive cells were detected in the grafts, showing the potential of NESC to differentiate into cerebellar neurons. However, we speculate that submitting NESC to a cerebellar phenotype induction^45^ before transplantation might enhance the differentiation into cerebellar neurons. Moreover, the neurotrophic factor levels were found significantly increased in the cerebellum of mice, as we have previously observed for murine NSC cerebellar transplantation^5^. Regarding interleukins, Control NESC transplantation promoted a more robust enhancement of the anti-inflammatory interleukin IL10 levels, whereas the MJD NESC increased the pro-inflammatory interleukin IL1B levels. These results might be explained by the described neuroinflammation activity of the mutant ataxin-3 protein^46,47^ present in the MJD NESC, which might be counteracting the natural anti-inflammatory activity of the neuroepithelial stem cells. Furthermore, cerebellar neurogenesis modulation was detected for both Control and MJD NESC through the increased levels of Msi1, an RNA-binding protein highly expressed in neural progenitors promoting the immature proliferating status of these cells^48^. Additionally, the Control NESC also enhanced the levels of NeuroD1, which is associated with neuronal differentiation and is required for the survival and maturation of adult-born neurons^49,50^. These results showed that both cell lines promote neurogenesis modulation and that Control NESC transplantation might result in the differentiation of more mature neurons, which has to be further investigated.

Finally, identifying markers to estimate from the established cell lines which ones present higher therapeutic potential would reduce the high cost and time-consuming in vivo testing of cell lines with no therapeutic potential. DLK1, EN2, and GBX2 genes have been previously studied as potential predictive markers of cell survival and differentiation after brain transplantation^51^. In the present work, the expression of DLK1, EN2, and GBX2 and also of MSI1, NEUROD1, and NOTCH1 genes, which we have previously used to evaluate the impact of anti-inflammatory therapies in neurogenesis^46^, were evaluated. Cell cultures obtained from MJD CLA NESC differentiation exhibited lower *EN2*, *MSI1*, and *NOTCH1* mRNA levels compared to CNT NESC. These results corroborate the in vitro and in vivo results showing that the MJD CLA NESC have inferior therapeutic potential, namely, MJD CLA NESC exhibited a lower ability to originate excitatory synapses both in vitro and in vivo, and after cerebellar transplantation promoted pro-inflammatory interleukin levels increase and triggered a less robust enhancement of neurogenesis-related proteins. Despite these encouraging data in the identification of predictive markers of cell therapeutic potential, further testing of *EN2*, *MSI1*, and *NOTCH1* mRNA quantification in other cell lines is required to evaluate whether the expression of these genes can be used to predict the cell therapeutic potency upon cerebellar transplantation.

In summary, the present study is a comprehensive evaluation of the therapeutic potential of human iPSC-derived NESC for the treatment of cerebellar diseases. Our data demonstrated that human iPSC-derived NESC upon transplantation differentiate into new neurons and maintain the bystander effects in the cerebellum for 6 months, which was observed even in cerebellar lobules far from the graft zone, indicating that a single injection of iPSC-derived NESC can significantly impact the cerebellum. As to the novelty, this study provides information regarding the therapeutic potential of human neuroepithelial stem cells generated from iPSC for cerebellar disease treatment. Moreover, we also performed an extensive evaluation of potential cell therapeutic mechanisms at 6 months after transplantation in the cerebellum. This, hopefully, adds knowledge to the cell therapy field, given the scarce information on the impact of cell transplantation, namely iPSC-derived NESC, in the cerebellum at later time points. As a limitation of this study, we point out that it is required to evaluate the human iPSC-derived NESC impact on the context of cerebellar disease. Namely, to assess whether these cells can improve the neuropathology and motor phenotype associated with these diseases.

## Materials and methods

### Ethics declarations

Human fibroblast isolation was performed under informed consent from all participants, was approved by the Ethics Committee of the Medical Faculty of the University of Coimbra, Portugal, and all research was performed in accordance with relevant guidelines/regulations.

All the experiments with animals were carried out in accordance with the European Community Council directive (86/609/ EEC) for the care and use of laboratory animals, were approved by the Responsible Organization for the Animals Welfare of the Faculty of Medicine and Center for Neuroscience and Cell Biology of the University of Coimbra and by the Portuguese Regulator (*DGAV-* 0421/000/000/2015) under ORBEA 66 (ORBEA_66_2015/22–06-2015), and all research was performed in accordance with relevant guidelines/regulations.

This study is reported in accordance with ARRIVE guidelines.

### Lentivirus production

The lentiviral vectors encoding for green fluorescent protein (GFP, SIN-PGK-GFP-WHV) and the lentivirus encoding for the 4 reprogramming factors^52^ were produced in 293 T HEK cells using a four-plasmid system, as previously described^53^. The lentiviral particles were stored at -80ºC and their concentration was determined by measuring the HIV-1 p24 antigen (RETROtek, Gentaur).

### Fibroblasts reprogramming into iPSC

Fibroblasts of one MJD patient (women, 31 years old, 79 CAG repetitions in the mutant ataxin-3 allele) and one Control (women, 52 years, no mutant allele) were previously established, and a normal diploid karyotype and genotyping indicating the number of CAG repetitions was also previously demonstrated^54^.

To reprogram human fibroblasts into iPSC, 10 000 fibroblasts growing in DMEM High-glucose (Sigma) medium supplemented with 10% FBS (Gibco), 1% Penicillin–Streptomycin (Gibco), 1% non-essential amino acids (Sigma), and 2 mM L-Glutamine (Gibco) were transduced with lentivirus (200 ng p24) encoding for the 4 reprogramming factors (c-Myc, Klf4, Sox2, and Oct4) and the dTomato fluorescent protein^52^. Fibroblasts were incubated with the virus for 12 h, then the culture medium was changed and cells were kept in this medium for 3 days. The lentivirus titration optimization in the fibroblasts used in this study has been performed by us^55^ according to a previously described study^56^. Transduction efficiency was evaluated in Control fibroblasts through the percentage of cells expressing dTomato (Supplementary Fig. S10). Afterward, fibroblasts were harvested via trypsinization, transferred onto mitomycin C-inactivated mouse embryonic fibroblast (MEF) feeders, and subsequently cultured in human pluripotent cell culture medium (DMEM F-12 (ThermoFisher), 20% Knock-out serum replacement (Gibco), 2 mM L-glutamine (Gibco), 1% nonessential amino acids, 100 μM β-mercaptoethanol, 1% Penicillin–Streptomycin, and 10 ng/mL of bFGF (Peprotech)). The culture medium was replaced every 1–3 days depending on the cell density. iPSC single colonies were picked based on morphology and self-silenced reprogramming factors (absence of dTomato)^52^. IPSC were split (1:4) by manual dissection and expanded on MEF feeder cells. Stable iPSC clones with at least 5 passages were established.

### iPSC-derived neuroepithelial stem cells (NESC) derivation

One Control iPSC clone and 3 MJD iPSC clones (from the same MJD patient) were then submitted to a neural induction protocol, as previously described^12^. Briefly, iPSC colonies were detached with 1 mg/mL dispase (STEMCELL Technologies), the cell clusters were collected through sedimentation and resuspended in human pluripotent cell culture medium (without bFGF) supplemented with 10 μM SB-431542 (Axon), 1 μM dorsomorphin (Sigma), 3.0 μM CHIR 99,021 (TargetMol), and 0.5 μM Purmorphamine (TargetMol) and cultured in non-treated MW6 plates. Then, the culture medium was replaced (50% on day 2, and 100% on day 3) by N2B27 medium (DMEM/F-12 no glutamine / Neurobasal (Gibco) 50:50, complemented with 1:200 N2 supplement (Gibco), 1:100 B27 supplement minus vitamin A (Gibco), 1% penicillin/streptomycin, and 1% L-glutamine) supplemented with 10 μM SB-431542, 1 μM dorsomorphin, 3.0 μM CHIR99021, and 0.5 μM Purmorphamine. On day 4, SB-431542 and dorsomorphin were withdrawn and 150 μM Ascorbic Acid (Sigma) was added to the medium. On day 6, the embryonic bodies showing neuroepithelial outgrowth were disrupted into smaller cell clusters and plated on Matrigel (hESC-qualified Matrix LDEV-Free, BD Matrigel, Corning) coated 12-well plates in NESC culture medium (N2B27 medium with 3.0 μM CHIR99021, 0.75 μM Purmorphamine, and 150 μM Ascorbic Acid). After a maximum of 5 splits, cultures were virtually free of contaminating non-NESC. One Control (CNT NESC) and three MJD (MJD CLA, MJD CLB, and MJD CLC NESC) cell lines were established and cultured in an incubator at 37ºC with 5% CO_2_. Frozen cell stocks were established in early passages (passage 5 to passage 10). Whenever needed cells were defrosted and cultured showing no change in morphology, proliferation, and differentiation capacity.

### Karyotype analysis

The karyotype analysis was performed by a certificated Genetic Laboratory (*Centro de Medicina Laboratorial Germano de Sousa*, Lisboa, Portugal) using standard G-banding techniques. Cells growing in a T25 flask covered with Matrigel were treated with 10 μg/ml Colcemid (0.05 μg/ml final) for up to 1 h, followed by dissociation with StemPro Accutase Cell Dissociation Reagent (Gibco). The cells were pelleted via centrifugation, re-suspended in pre-warmed 0.075 M KCl hypotonic solution, and incubated for 5 min at 37ºC. Following centrifugation, the cells were re-suspended in Carnoy’s fixative (3:1 ratio of methanol: glacial acetic acid). Metaphase spreads were prepared on glass microscope slides and GTG-banded by brief exposure to trypsin and stained with Leishman. Fifty metaphases were analyzed for each cell line and karyotypes were established according to the International System for Human Cytogenetic Nomenclature (ISCN) 2016.

### iPSC-derived NESC culture and differentiation

Cells growing in monolayers in Matrigel-coated cell culture flasks cultured in NESC culture medium, as previously described, were detached with pre-warmed StemPro Accutase Cell Dissociation Reagent for 3–5 min at 37ºC. Then, cells were diluted in KnockOut DMEM (Gibco) and precipitated through centrifugation at 1100 RPM for 5 min. For cell differentiation in neural cultures composed of neurons and glia, 250 000 cells/well (MW12 plates) were plated on Matrigel-coated coverslips in N2B27 medium supplemented with 250 μM Dibutyryl cyclic-AMP sodium salt (dbcAMP) (BIOLOG), 5 μM Forskolin (TargetMol), and 2 μM retinoic acid (Sigma) as previously reported^5,46^. Cells were maintained for up to 2 months and the culture medium was changed every five days.

### Human brain organoids establishment

Brain organoids were obtained as previously described^19^. Briefly, Control and MJD iPSC-derived NESC cultured in N2B27 medium complemented with 3.0 μM CHIR99021, 0.75 μM Purmorphamine, and 150 μM Ascorbic Acid were plated at 9000 cells/well/100 µL in an ultra-low attachment 96-well flat-bottom plate (Corning Costar). This culture medium was added every other day for 6 days. Then, spheroids were transferred to a 24-well ultra-low attachment flat-bottom plate (Corning Costar). Two days later (day 8), the spheroids were transferred to cold droplets of Matrigel and cultured in the same culture medium for 2 days. On day 10, N2B27 medium supplemented with 10 ng/mL human BDNF, 10 ng/mL human GDNF, 1 ng/mL human TGFβ3, 500 μM dbcAMP, 200 μM ascorbic acid, and 1 μM purmorphamine was used to induce differentiation. On day 14, organoids were placed on an orbital shaker (65 rpm), in an incubator at 37ºC with 5% CO_2_. From day 16, the culture medium was changed every 3–4 days using N2B27 medium supplemented with 10 ng/mL BDNF, 10 ng/mL GDNF, 1 ng/mL TGFβ-3, 500 μM dbcAMP, and 200 μM ascorbic acid. Organoids were kept in these culture conditions until the end of the experiment.

### GFP expression and maturation of iPSC-derived NESC prior to cerebellar transplantation

NESC plated in T75 flasks covered with Matrigel were infected with lentivirus encoding for GFP as previously reported^5^. Briefly, 3 × 10^6^ cells were infected with lentivirus (1.33 × 10^3^ ng of p24 antigen) and 24 h later half of the culture medium was replaced to dilute the virus. On the second day, the lentiviral vectors were completely removed by changing the medium. Five days before transplantation, cells were submitted to a maturation protocol with the N2B27 medium supplemented with 10 ng/ml human BDNF (Peprotech), 10 ng/ml human GDNF (Peprotech), 1 ng/ml human TGFβ-3 (Peprotech), 200 μM Ascorbic Acid, and 250 μM dbcAMP. For transplantation, cells were prepared in Hank's Balanced Salt Solution HBSS (Sigma) at 150 000 cells/2 μl.

### Immunocytochemistry

Cells in coverslips were washed two times with PBS, fixed with 4% paraformaldehyde (PFA) (Sigma) for 20 min at room temperature, washed with PBS, and stored at 4ºC until further processing. Then, cells were permeabilized for 5 min with 1% Triton X-100 (Sigma), washed three times with PBS, and blocked in 3% bovine serum albumin (BSA) (Sigma) in PBS for 1 h at room temperature. Cells were then incubated overnight at 4ºC with primary antibodies (MAP2, GFAP, β3 Tubulin, S100B, and O4; Supplementary Table S1) diluted in 3% BSA in PBS. Afterward, coverslips were washed two times with PBS and incubated for 2 h at room temperature with secondary antibodies Alexa Fluor-594 anti-mouse and Alexa Fluor-488 anti-rabbit (1:200, Invitrogen) diluted in 1% BSA in PBS. Subsequently, cells were washed two times with PBS; nuclei were stained with 4′, 6-diamidino-2-phenylindole (DAPI) (1:5000, Applichem) for 5 min, and then washed 3 times with PBS. Finally, coverslips were mounted with a fluorescence mounting medium (Dako). Images were acquired at room temperature with an Axio Imager Z2 widefield microscope (CCD monochromatic digital camera Axiocam HRm) using EC Plan-Apochromat 10x/0.3NA or Plan-Apochromat 20x/0.8NA air objectives.

### Total neurite length measurement and synapses quantification in cell cultures

Cells plated in coverslips, as previously described, were washed two times with PBS, fixed with 4% PFA in PBS for 20 min at room temperature, washed again with PBS, and stored at 4ºC until further processing. Then, cells were permeabilized for 5 min in 0.25% Triton X-100 at room temperature, washed three times with PBS, and blocked in 10% BSA in PBS for 1 h. Primary antibodies (VGluT1, PSD95, MAP2, β3 Tubulin, and Gephyrin; Supplementary Table S2) were prepared in 3% BSA in PBS and centrifuged for 10 min at 13,000 rpm, 4ºC, to remove antibody aggregates. Cells were incubated overnight with the primary antibodies. Afterward, cells were washed two times with PBS, followed by a 45 min incubation at 37ºC with Alexa Fluor conjugated secondary antibodies, diluted in 1% BSA in PBS. Coverslips were then washed two times with PBS, nuclei were stained with DAPI (1:5000) for 5 min, washed 3 times with PBS, dried, and mounted with fluorescence mounting medium (Dako). Images were acquired with an Axio Imager Z2 widefield microscope (CCD monochromatic digital camera Axiocam HRm). For neurite length measurement it was used EC Plan-Apochromat 10x/0.3NA or Plan-Apochromat 20x/0.8NA air objectives after randomly selecting MAP2-positive cellular tracts. For synapse quantification it was used a Plan-Apochromat 63x/1.4NA oil objective after randomly selecting MAP2/ β3 Tubulin-positive cellular tracts. At least five pictures were acquired and analyzed from each condition. Images were analyzed blindly to condition using Fiji software. The number of colocalized puncta or the total number of inhibitory postsynaptic terminals were normalized per dendritic section length.

### Western blot

Cells were disrupted using a lysis buffer (150 mM NaCl, 50 mM Tris, 5 mM EDTA, 1% Triton X-100, 0.5% sodium deoxycholate, and 0.1% SDS added freshly with protease inhibitor (cOmplete Mini, Roche), phosphatase inhibitor (PhosStop, Roche), 1 mM Phenylmethane Sulfonyl Fluoride (PMSF) (Sigma-Aldrich), and 10 μg/mL Dithiothreitol (DTT) (Sigma-Aldrich)) and a strong vortex followed by 3 sonication cycles of 10 s (40 kHz). The amount of protein in each sample was quantified using Pierce BCA Protein Assay Kit (Thermo Fischer Scientific) and 50 μg of protein per sample was loaded. Samples were prepared with sample buffer (0.5 M Tris–HCl, pH 6.8, 30% glycerol, 10% SDS, 0.6 M DTT, and 0.1 mg/mL blue bromophenol), denatured at 95ºC for 5 min, and stored at -20ºC until use. Proteins were separated by SDS–polyacrylamide gel electrophoresis, in 10% resolving acrylamide gels at 70 V for 10–15 min and 100 V for 45—60 min. Afterward, proteins were transferred into polyvinylidene difluoride (PVDF) membranes (Immobilon-P Membrane, Millipore). The transference was done at 0.75 A and 4ºC for 2 h and 30 min. Then, membranes were blocked with 5% milk in Tris-Buffered Saline (TBS) with 0.1% Tween 20 (TBS-T) for 1 h. Incubation with primary antibodies (Tra-1–60, Pax6, ataxin-3, and Actin; Supplementary Table S2), prepared in 5% milk in TBS-T, was done overnight at 4ºC. Subsequently, membranes were washed three times with TBS-T and incubated with alkaline phosphatase-linked secondary antibodies for 2 h at room temperature. Membranes were washed with TBS-T three times and proteins were detected using Enhanced Chemifluorescence substrate (ECF, Amersham Biosciences) in the Chemidoc (Bio-Rad) and analyzed in Image Lab software (Bio-Rad).

### Reverse transcription-quantitative real-time polymerase chain reaction (RT-qPCR)

NESC cultures and organoids (a 3–5 organoids pool depending on the time point/organoids’ size) were washed 3 times with PBS and kept at -80ºC until processing. RNA was extracted using the NucleoSpin RNA kit (Macherey–Nagel) according to the manufacturer’s instructions. The RNA purity and concentration were measured with NanoDrop 2000 (Thermo Scientific). Then, 1000 ng of total RNA was converted into cDNA with iScript cDNA Synthesis Kit, according to the manufacturer’s instructions (Bio-Rad). Quantitative real-time PCR was performed with the SsoAdvanced SYBR Green Supermix Kit (Bio-Rad) using cDNA diluted 10 times with DNase-free water (Sigma) and the protocol: a single cycle of 95ºC for 30 s followed by 45 cycles of two steps, one step of 5 s at 95ºC followed by a step of 15 s at a temperature depending on each primer set (Supplementary Table S3). StepOne Software (Applied Biosystems) generated automatically a threshold cycle (CT) for each gene. In each experiment, and for all genes, a standard curve was done, and no template and no reverse transcriptase controls were performed. Subsequently, the software determined the PCR amplification efficiency and R^2^. The mRNA relative quantification with respect to control samples was determined by the Pfaffl method taking into consideration the different amplification efficiencies of all genes.

### Metabolic activity

Metabolic activity was assessed by the resazurin reduction assay. For this, a single organoid/well (in 96-well plates) was incubated with 0.1 mg/mL resazurin, prepared in the organoids culture medium previously described, for 4 h at 37ºC and 5% CO_2_. The absorbance for the reduced and oxidized species of resazurin was read at 570 nm and 600 nm, respectively, using a spectrophotometer (SpectraMax Plus 384, Molecular Devices). Metabolic activity was evaluated through absorbance at 570 nm subtracted from 600 nm absorbance.

### Single-cell calcium imaging

For cells plated in coverslips, the intracellular calcium concentration changes were evaluated upon potassium and histamine depolarization, markers of functional differentiated neurons and undifferentiated neural progenitors, respectively^5,17^. For brain organoids, cultured as previously described, the intracellular calcium concentration changes were evaluated upon potassium depolarization. Cells and organoids were washed twice in 0.1% BSA/Krebs buffer (142 mM NaCl, 1 mM KCl, 1 mM MgCl_2_, 10 mM Glucose, 10 mM HEPES, 10 mM NaHCO_3,_ and 1 mM CaCl_2_, pH 7.4) and incubated with 5 μM Fluo-4/AM (a calcium indicator, increasing its fluorescence excitation at 488 nm upon Ca^2+^ binding, thus increasing the fluorescence signal) (Invitrogen) and 0.02% pluronic acid in 0.1% BSA/Krebs buffer for 45 min at 37ºC. Afterward, cells and organoids were transferred to 0.1% BSA/Krebs buffer and kept at 37ºC during microscopy observation. For observation, the basal fluorescent levels were measured for 4 min. Then, the depolarization stimulus, 50 mM KCl/Krebs (96 mM NaCl, 50 mM KCl, 1 mM MgCl_2_, 10 mM Glucose, 10 mM HEPES, 10 mM NaHCO_3,_ and 1 mM CaCl_2_, pH 7.4) for cells, and 50 mM KCl in 0.1% BSA/Krebs buffer for organoids, was added for 2 min, followed by a 4-min repolarization period in 0.1% BSA/Krebs. Subsequently, for the cell cultures, the second depolarization stimulus (100 μM histamine in 0.1% BSA/Krebs buffer) was added for 2 min, followed again by a 4-min repolarization period in 0.1% BSA/Krebs buffer. Fluorescence measurement at 488 nm was performed at 37ºC and continuously for all the experiments with a Carl Zeiss Cell Observer Spinning Disk microscope (EM-CCD Evolve Delta camera) using a Plan-Apochromat 20x/0.8NA air objective. Image analysis was performed in Fiji, where cell bodies were drawn and the shift in signal intensity was measured. Cells responding to potassium were identified as functional neurons, while cells responding to histamine were identified as neural progenitors. Cells were considered to respond to the potassium stimulus when a 15% increase in signal (Ca^2+^ concentration) was observed, while cells presenting a histamine/potassium ratio equal to or above 1 were considered to respond to histamine and consequently considered as neural progenitors as previously reported^5^. The response amplitude for each neuron was determined by the maximum fluorescence value normalized by the basal fluorescence.

### Organoids sectioning and immunolabeling

Brain organoids were collected on day 40, washed three times in PBS, and fixed with 4% PFA for 20 min at 4ºC. Then, organoids were washed three times with PBS and kept in 30% sucrose in PBS for 24 h at 4ºC. Organoids were subsequently frozen at -80ºC in Tissue-Tek O.C.T. Compound (Sakura) and kept at -80ºC until further sectioning. Organoids were sliced at 35 μm using a cryostat (Thermo Fisher Scientific) and collected in Superfrost Plus slides (Thermo Scientific). Slides were dried at 37ºC for 25 min and kept at 4ºC until immunohistochemistry processing. Afterward, sections were washed three times with PBS and blocked and permeabilized with 0.1% Triton X-100 and 10% normal goat serum (NGS) in PBS, for 2 h at room temperature. Then, sections were incubated overnight at 4ºC with the primary antibodies (Nestin, GFAP, MAP2, PCP4, Ataxin-3, Msi1, and NeuroD1; Supplementary Table S2) prepared in 10% NGS in PBS. Subsequently, sections were washed three times with PBS and incubated with the Alexa Fluor conjugated secondary antibodies, prepared in 2% NGS in PBS, for 2 h at room temperature. The nuclei were stained with DAPI (1:5000) for 10 min at room temperature. Sections were then washed three times with PBS, dried, and mounted with Mowiol reagent (Sigma). Fluorescence images were acquired with a Plan-Apochromat 20x/0.8NA air objective on an Axio Imager Z2 widefield microscope.

### Organoids’ size

Sections from organoids collected as previously described were washed three times with PBS before incubation with the nuclear DNA staining reagent DAPI for 10 min. The sections were then washed three times with PBS, air dried, and mounted with Mowiol reagent (Sigma). Widefield fluorescence images were acquired with an EC Plan-Apochromat 20x/0.8NA air objective. A tile image was acquired and stitched together to reconstitute the entire organoid. ImageJ/Fiji software was used to determine the organoids’ area in the DAPI channel.

### Stereotaxic injection of iPSC-derived NESC

For in vivo experiments, immunodeficient NOD.CB17-Prkdc scid/J female mice (NOD.SCID) (Charles River) were used. In each experiment, littermates with similar weights were caged randomly and submitted to 7 days of acclimatization before the stereotaxic surgery. The NOD.SCID (postnatal days 42–46) were submitted to deep anesthesia (ketamine/medetomidine) and received a single stereotaxic injection of 150 000 cells prepared in 2 μl HBSS (Sigma) injected in lobule 5 of the cerebellum at a rate 0.25 μl/min with an automatic injector (Stoelting Co., Wood Dale, USA) at the following coordinates: anteroposterior: − 2.3 mm, lateral: 0 mm and ventral: − 3.0 mm, relative to lambda. For the 2-month-time point the following number of animals were transplanted: CNT NESC n = 4, MJD CLA NESC n = 3, MJD CLB n = 5, and MJD CLC NESC n = 4 mice. For the 6-month-time point CNT NESC n = 5, MJD CLA NESC n = 3, and HBSS-injected n = 4 mice were transplanted. Mice were housed in sterile conditions (Ventiracks) and food and water were provided ad libitum*.*

### Mice brain tissue preparation and immunohistochemistry

After induction of deep anesthesia (ketamine/medetomidine) mice were intracardiacally perfused with 4% cold PFA, brains were removed, post-fixed in 4% PFA for 24 h at 4ºC, and cryoprotected in 25% sucrose in PBS for 48 h at 4ºC. Then, brains were frozen at -80ºC and 25 μm-sagittal sections were cut using a cryostat (Thermo Fisher Scientific). Brain slices were collected in 48-well plates as free-floating sections in PBS/0.05 mM sodium azide and were stored at 4ºC until processing. Brain sections (two sections per animal) were washed three times with PBS and blocked and permeabilized with 10% NGS and 0.1% Triton X-100 in PBS for two hours. Primary antibodies (GFAP, MAP2, Iba1, cleaved Caspase-3, Ki67, PCP4, Calbindin, Parvalbumin, GABA, Synaptophysin, Gephyrin, PSD95, VGluT1, NGF, GDNF, IL10, IL1B, IL6, Msi1, and NeuroD1; Supplementary Table S2) were diluted in 10% NGS and 0.05% Triton X-100 in PBS and incubated overnight at 4ºC. For synapses quantification the diluted antibodies were centrifuged at 4ºC for 10 min at 13,000 rpm prior to incubation, to remove antibody aggregates. Then, after three washes in PBS, brain sections were incubated for two hours with Alexa Fluor conjugated secondary antibodies (1:200, Invitrogen) diluted in 2% NGS in PBS. Sections were nuclei stained with DAPI (1:5000) for 10 min and washed three times in PBS. Brain sections were mounted in glass slides covered with gelatin, left to dry for 15 min at 37ºC, and mounted with Mowiol mounting medium. Widefield fluorescence images were acquired with an Axio Imager Z2 microscope (CCD monochromatic digital camera Axiocam HRm) using EC Plan-Apochromat 10x/0.3NA or Plan-Apochromat 20x/0.8NA air objectives. Confocal fluorescence images were obtained with an LSM 710 Axio Observer using Plan-Apochromat 20x/0.8NA air objective and Plan-Apochromat 63x/1.4NA oil objective. For synapse quantification in graft-derived neurons, Z-stack confocal microscopy pictures were acquired with the 63x/1.4NA Plan-ApoChromat oil objective with exposure times conserved in the experiments. IMARIS software (Oxford Instruments) was used to obtain the 3D representation of the confocal microscopy pictures and to quantify synapses as pre/post-synaptic spots adjacent to each other. For immunofluorescence quantification, two sections (immediately next to the cerebellum sections where the cell grafts were detected but not containing human cells) were analyzed. Exposure times were conserved in the experiments, tiff images (obtained from microscopy pictures) were processed using ImageJ/FIJI (National Institutes of Health), and mean intensity was obtained for each lobule(s) quantified (lobule 4 and 5 (Lobules 4–5), lobule 6, 7 and 8 (lobules 6–8), Lobule 9, and Lobule 10).

### Quantification of undifferentiated and differentiated human iPSC-derived NESC

For in vivo cell differentiation, immunofluorescence Z-stack pictures of the whole human graft in cerebellar sections were acquired with a Plan-Apochromat 40 × /1.40 Oil DIC M27 objective on a Zeiss LSM 710 confocal microscope. Zeiss Zen software was used to obtain orthogonal reconstructions of the confocal z-stack pictures. Then, ImageJ software was used for the quantification of positive cells for each specific cell marker. The threshold of each channel was adjusted so that only positive staining remained. Following this, regions of interest (ROI) corresponding to the human nuclei (HuNu-positive cells) were selected, and these ROIs were placed on the channel of the cell marker of interest (Pax6, β3 Tubulin, NeuN, S100B). The colocalization percentage between both channels was then measured, and cells exhibiting more than 10% colocalization were considered positive for the marker. For in vitro cell differentiation, four immunofluorescence pictures were randomly acquired for each condition of each of the four independent experiments with a Zeiss LSM 710 confocal microscope using a Plan-Apochromat 20x/0.8NA air objective. The quantification method was equal to the previously described for in vivo cell differentiation, except that the regions of interest (ROI) corresponding to the human nuclei were established on the DAPI channel, which was then placed on the channel of the cell marker of interest (MAP2 and GFAP). The colocalization percentage between both channels was then measured, and cells exhibiting more than 8% colocalization were considered positive for the marker.

### Cresyl violet staining and quantification of cerebellum total area and cellular layers area

Sections were pre-mounted and stained as previously described^5^. Quantification of molecular and granular cerebellar layers area as well as cerebellum total area was made over six sections per animal. Brightfield images were acquired with an Axio Scan.Z1 microscope using a Plan-Apochromat 20x/0.8 M27 air objective. For quantification, the ImageJ software (NIH, USA) was used.

### Statistical analysis

All data are presented as mean ± SEM. Graphs and statistical analysis were performed in Graph Pad Prism 6 software. Statistical significance was assessed by unpaired t-test and One-way ANOVA; values of *p* < 0.05 were considered statistically significant (*p < 0.05, **p < 0.01, ***p < 0.001, and ****p < 0.0001 as indicated in the legend of the figures).

### Supplementary Information

Supplementary Information.

## Data Availability

The data and materials are available from the corresponding authors upon reasonable request, but we may require a payment and/or a completed materials transfer agreement if there is potential for commercial application.
