# Supplementary material for: Graft-derived neurons and bystander effects are maintained for six months after human iPSC-derived NESC transplantation in mice’s cerebella
Source: Sci Rep. 2024 Feb 8;14:3236. doi: 10.1038/s41598-024-53542-x (PMC10853537; doi:10.1038/s41598-024-53542-x)

**Graft-derived neurons and bystander effects are maintained for six months after human iPSC-derived NESC transplantation in mice’s cerebella**

Liliana S. Mendonça^1, 2, 3*^, Daniel Henriques^1, 2, 3^, Vanessa Fernandes^1^, Ricardo Moreira^1,^ ^2, 4^, João Brás^1^, Sónia Duarte^1, 2, 3^, Jens C. Schwamborn^5^, Luís Pereira de Almeida^1, 2, 4*^

1. Center for Neuroscience and Cell Biology, University of Coimbra, Coimbra, Portugal.

2. Center for Innovative Biomedicine and Biotechnology, University of Coimbra, Coimbra, Portugal.

3. Institute of Interdisciplinary Research, University of Coimbra, Coimbra, Portugal

4. Faculty of Pharmacy, University of Coimbra, Coimbra, Portugal.

5. Luxembourg Centre for Systems Biomedicine, University of Luxembourg, Belvaux, Luxembourg.

Correspondence: Liliana Mendonça* liliana.mendonca@cnc.uc.pt and Luís Pereira de Almeida* luispa@ci.uc.pt.

**Supplementary Tables**

**Table S1: Characterization of iPSC-derived NESC**

| Cell line^a^ | Before differentiation | | | Derived neural cultures (3 weeks of *in vitro* differentiation) | | | | |
| --- | --- | --- | --- | --- | --- | --- | --- | --- |
|  | “NESC like” | Karyotype | Mt Atx3 | Neurons  and glia | Neurites^b^ | Exc. synapses^b^ | Inhi. terminals^b^ | Firing neurons (%) |
| CNT | OK | OK | N.o. | Present | 1.000 | 1. 000 | 1. 000 | 85.85 |
| CLA | OK | X | Present | Present | 1.038 | 0.614 | 1.112 | 93.70 |
| CLB | OK | OK | Present | Present | 0.815 | 0.839 | 0.992 | 90.97 |
| CLC | OK | OK | Present | Present | 1.236 | 1.067 | 1.195 | 65.42 |

^a^ iPSC-derived NESC: CNT (control), CLA (MJD CLA NESC), CLB (MJD CLB NESC), and CLC (MJD CLC NESC); NESC “like”: OK = characteristic morphology of NESC growing in monolayer and exhibiting self-renewal and expansion; Karyotype: OK = normal chromosomal constitution, X = abnormal chromosomal constitution; Mt Atxn3: N.o. = mutant ataxin-3 not observed, Present = mutant ataxin-3 observed; ^b^ relative ratio (normalized for CNT cells); Exc. synapses: Excitatory synapses; Inhi. terminals: inhibitory post-synaptic terminals.

**Table S2: Antibodies**

| Antibody | Brand | Reference | Host | Dilution |
| --- | --- | --- | --- | --- |
| Actin | Sigma | AP4G5 | Mouse | 1:1000 |
| Ataxin-3 | Millipore | MAB5360 | Mouse | 1:1000 |
| β3 Tubulin | Invitrogen | 480011 | Mouse | 1:500 |
| Calbindin | Millipore | AB1778 | Rabbit | 1:1000 |
| cleaved  Caspase-3 | Cell Signaling | 9661 | Rabbit | 1:1000 |
| GABA | Sigma-Aldrich | A2052 | Rabbit | 1:1000 |
| GDNF | Santa Cruz Biotechnology | sc-13147 | Mouse | 1:100 |
| Gephyrin | Synaptic Systems | 147011 | Mouse | 1:500 |
| GFAP | DAKO | Z0334 | Rabbit | 1:400 |
| Human Nuclei (HuNu) | Millipore | MAB1281 | Mouse | 1:500 |
| Iba1 | WAKO | 019-19741 | Rabbit | 1:500 |
| IL1B | Santa Cruz Biotechnology | sc-52012 | Mouse | 1:500 |
| IL6 | Santa Cruz Biotechnology | sc-32296 | Mouse | 1:500 |
| IL10 | Santa Cruz Biotechnology | Sc-8438 | Mouse | 1:500 |
| Ki67 | Abcam | ab16667 | Rabbit | 1:500 |
| MAP2 | Sigma-Aldrich | M1406 | Mouse | 1:250 |
| Msi1 | Abcam | ab21628 | Rabbit | 1:500 |
| Nestin | R&D systems | MAB1259 | Mouse | 1:250 |
| NeuroD1 | Abcam | ab60704 | Mouse | 1:300 |
| NGF | Santa Cruz Biotechnology | sc-365944 | Mouse | 1:500 |
| O4 | R&D systems | MAB1326 | Mouse | 1:500 |
| Parvalbumin | Sigma-Aldrich | P3088 | Mouse | 1:1000 |
| Pax6 | (Novex) Life Technologies | 42-6600 | Rabbit | 1:250 |
| PCP4 | Santa Cruz Biotechnology | sc-74816 | Rabbit | 1:100 |
| PSD95 | Cell Signaling | 3450 | Rabbit | 1:1000 |
| S100B | Abcam | Ab52642 | Rabbit | 1:500 |
| Synaptophysin | Millipore | AB9272 | Rabbit | 1:1000 |
| Tra-1-60 | Invitrogen | 41-1000 | Mouse | 1:500 |
| VGlut1 | Millipore | AB5905 | Guinea pig | 1:1000 |

**Table S3: Primers for RT-qPCR**

| **Gene** | **Brand** | **Sequence /reference** | **Annealing temperature** |
| --- | --- | --- | --- |
| TUBB3 | Invitrogen | Fwd: GGCCAAGGGTCACTACACG  Rev: GCAGTCGCAGTTTTCACACTC | 58ºC |
| PAX6 | Sigma | KiCqStart Pre-designed Primers | 58ºC |
| MAP2 | Invitrogen | Fwd: CGAAGCGCCAATGGATTCC  Rev: TGAACTATCCTTGCAGACACCT | 57ºC |
| GFAP | Invitrogen | Fwd: AGGTCCATGTGGAGCTTGAC  Rev: GCCATTGCCTCATACTGCGT | 58ºC |
| NEFL | Sigma | KiCqStart Pre-designed Primers | 57ºC |
| GBX2 | Sigma | KiCqStart Pre-designed Primers | 58ºC |
| EN2 | Sigma | KiCqStart Pre-designed Primers | 58ºC |
| DLK1 | Sigma | KiCqStart Pre-designed Primers | 59ºC |
| BDNF | Sigma | KiCqStart Pre-designed Primers | 58ºC |
| MSI1 | Sigma | KiCqStart Pre-designed Primers | 60ºC |
| NEUROD1 | Sigma | KiCqStart Pre-designed Primers | 58ºC |
| NOTCH1 | Sigma | KiCqStart Pre-designed Primers | 60ºC |
| HPRT | Qiagen | Hs_HPRT1_1_SG QuantiTect Primer Assay, QT00059066 | 60ºC |

**Supplementary Figures**

**
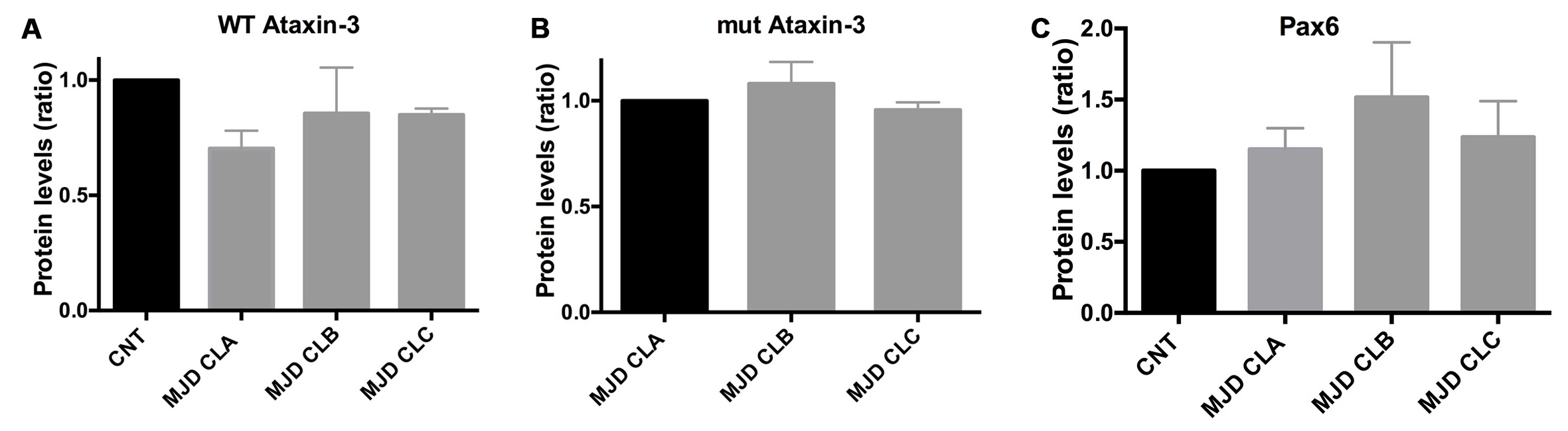
 Supplementary Fig. S1. Quantification of Pax6 and wild type and mutant Ataxin-3 protein levels in human iPSC-derived NESC. A)** Wild-type (WT) and (**B**) mutant (mut) Ataxin-3 and (**C**) Pax6 protein levels of Control (CNT), MJD CLA, MJD CLB, and MJD CLC iPSC-derived NESC (MJD CLA, MJD CLB, and MJD CLC, respectively) evaluated by western-blot assay; protein levels were normalized for actin and CNT, n=3 independent experiments. Data are expressed as mean ± SEM, One-way ANOVA followed by Tukey’s post-test.


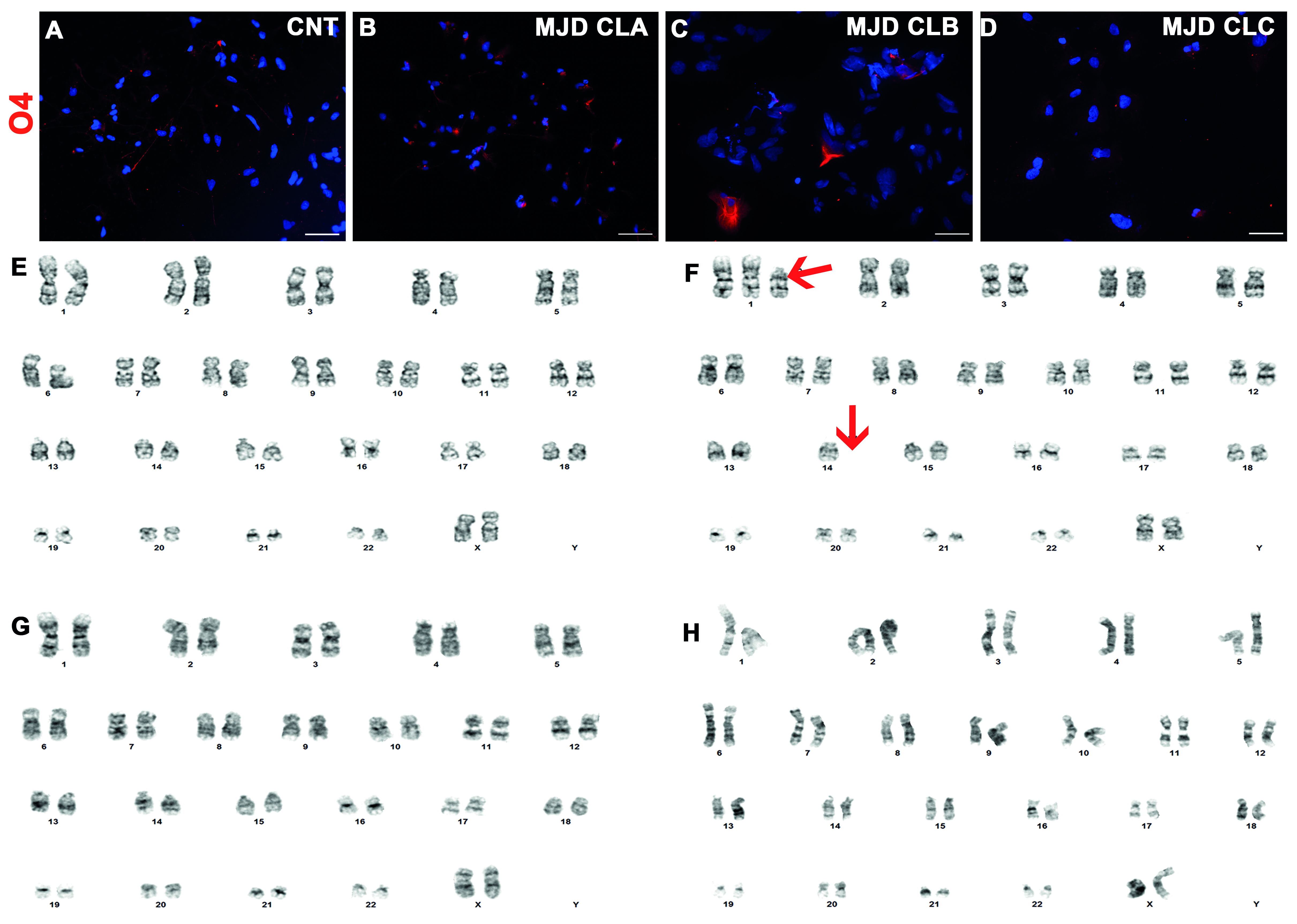
**Supplementary Fig. S2. Evaluation of oligodendrocytes presence in Control and MJD iPSC-derived NESC differentiated cultures and cells’ karyotype stability. (A** - **D)** Representative fluorescence images of CNT and MJD iPSC-derived NESC differentiated cultures immunolabeled for the oligodendrocyte marker O4. O4-positive cells were only detected in a few cells of the MJD CLB NESC cultures. DAPI: blue, representative images of 3 independent experiments, scale bars: 50 μm. Karyotype analysis demonstrated **(E**, **G** and **H)** a normal diploid karyotype for CNT, MJD CLB, and MJD CLC iPSC-derived NESC (nomenclature ISCN 2016: 46,XX), whereas **(F)** MJD CLA iPSC-derived NESC exhibited a reciprocal and unbalanced translocation between the long arms of chromosome 1 and chromosome 14 (red arrows), which resulted in a partial trisomy of the long arm of chromosome 1 and partial monosomy of the long arm of chromosome 14 (nomenclature ISCN 2016: 46,XX,der(14?)t(1;14)(q12;q13)).


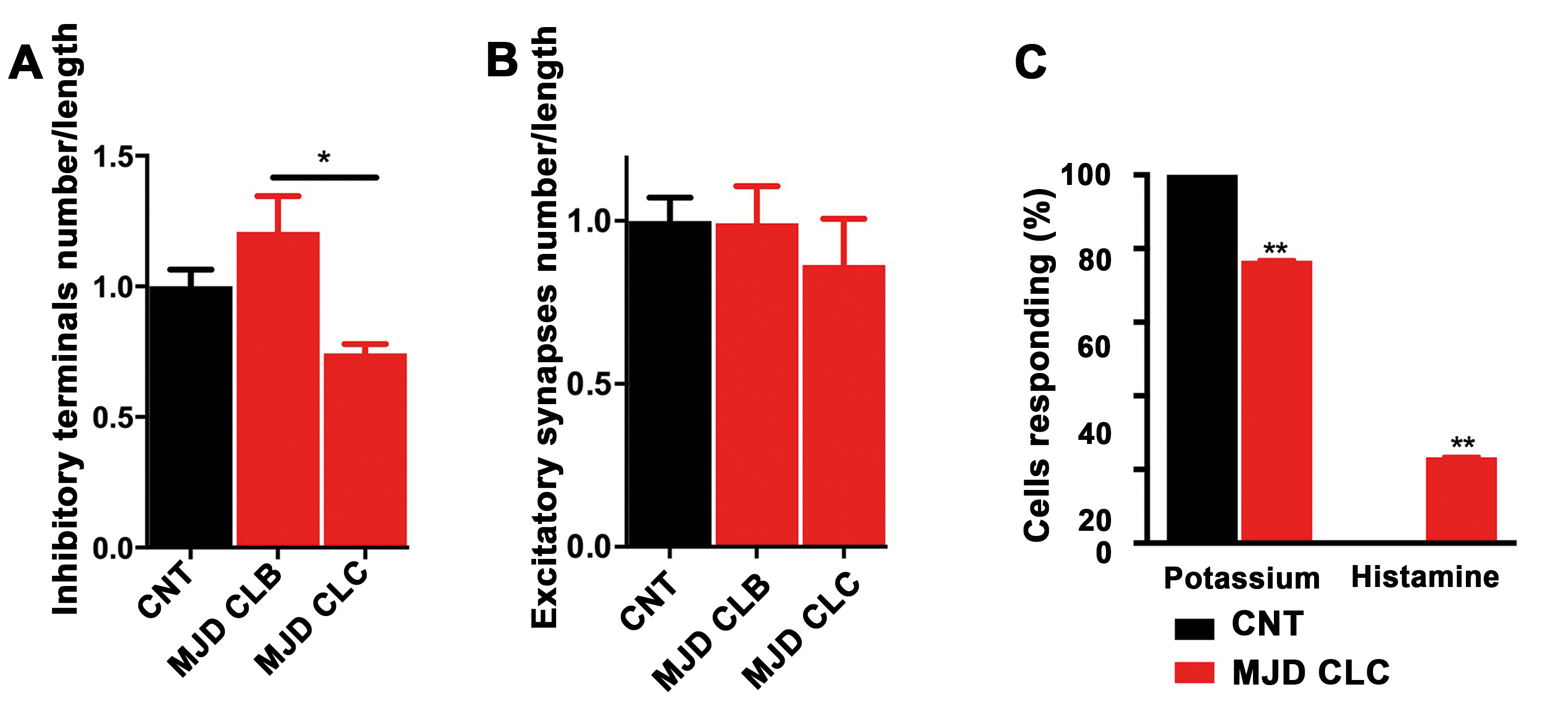
**Supplementary Fig. S3. Evaluation of neuronal firing and synapses number in neurons after two months of differentiation.** Control, MJD CLA, MJD CLB, and MJD CLC iPSC-derived NESC (CNT, MJD CLA, MJD CLB, and MJD CLC, respectively) were differentiated into neuronal cultures for 2 months. (**A**) Number of inhibitory postsynaptic terminals per neurite length normalized for CNT NESC (n=3 independent experiments; analyzed neurons: CNT n=41, MJD CLB n=47, MJD CLC n=39). (**B**) Number of excitatory synapses per neurite length normalized for CNT NESC (n=3 independent experiments; analyzed neurons: CNT n=38, MJD CLB n=38, MJD CLC n=44). **(C)** Neuronal firing evaluation through the variation of intracellular calcium concentration in neurons with single-cell calcium imaging. Percentage of cells responding to potassium (neurons) and histamine (neural progenitors) stimulus evaluated in cultures differentiated for 2 months (n = 3-5 independent experiments, analyzed cells: CNT n=95, MJD CLC n=69). Data are expressed as mean ± SEM, *p<0.05 and **p<0.01, (A-B) One-way ANOVA followed by Tukey’s post-test, and (C) unpaired t-test with Welch's correction.


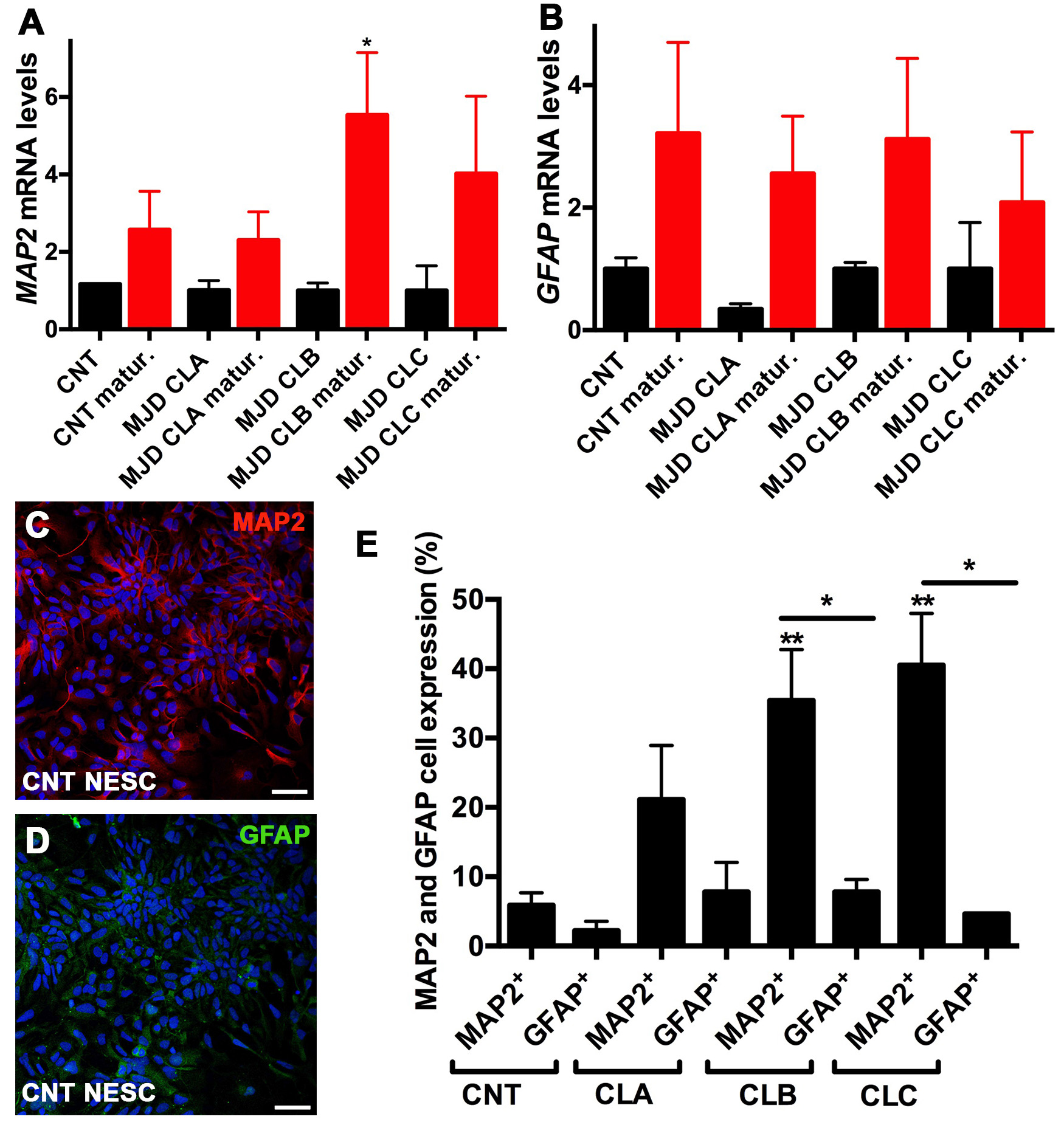


**Supplementary Fig. S4. Evaluation of MAP2 and GFAP expression in the iPSC-derived NESC submitted to maturation protocol before cerebellar transplantation. A)** *MAP2* and **B)** *GFAP* mRNA levels of CNT, MJD CLA, MJD CLB, and MJD CLC iPSC-derived NESC after cell maturation protocol (CNT matur., MJD CLA matur., MJD CLB matur., and MJD CLC matur., respectively) assessed by RT-qPCR and normalized for mRNA levels of CNT, MJD CLA, MJD CLB, and MJD CLC iPSC-derived NESC (CNT, MJD CLA, MJD CLB, MJD CLC, respectively) without cell maturation; CNT, CLA, CLB n=4 and CLC n=2 independent experiments. (**C-E**) MAP2 and GFAP expression in iPSC-derived NESC submitted to cell maturation assessed by immunocytochemistry. Representative fluorescence microscopy images showing (**C**) MAP2 and (**D**) GFAP expression in Control iPSC-derived NESC (CNT NESC) submitted to cell maturation; scale bars: 50 μm. (**E**) MAP2 (MAP2^+^) and GFAP (GFAP^+^) cell expression (%) in CNT, MJD CLA, MJD CLB, and MJD CLC iPSC-derived NESC (CNT, CLA, CLB, and CLC, respectively) upon cell maturation; n=4 independent experiments. Data are expressed as mean ± SEM, One-way ANOVA followed by Tukey's multiple comparisons post-test.


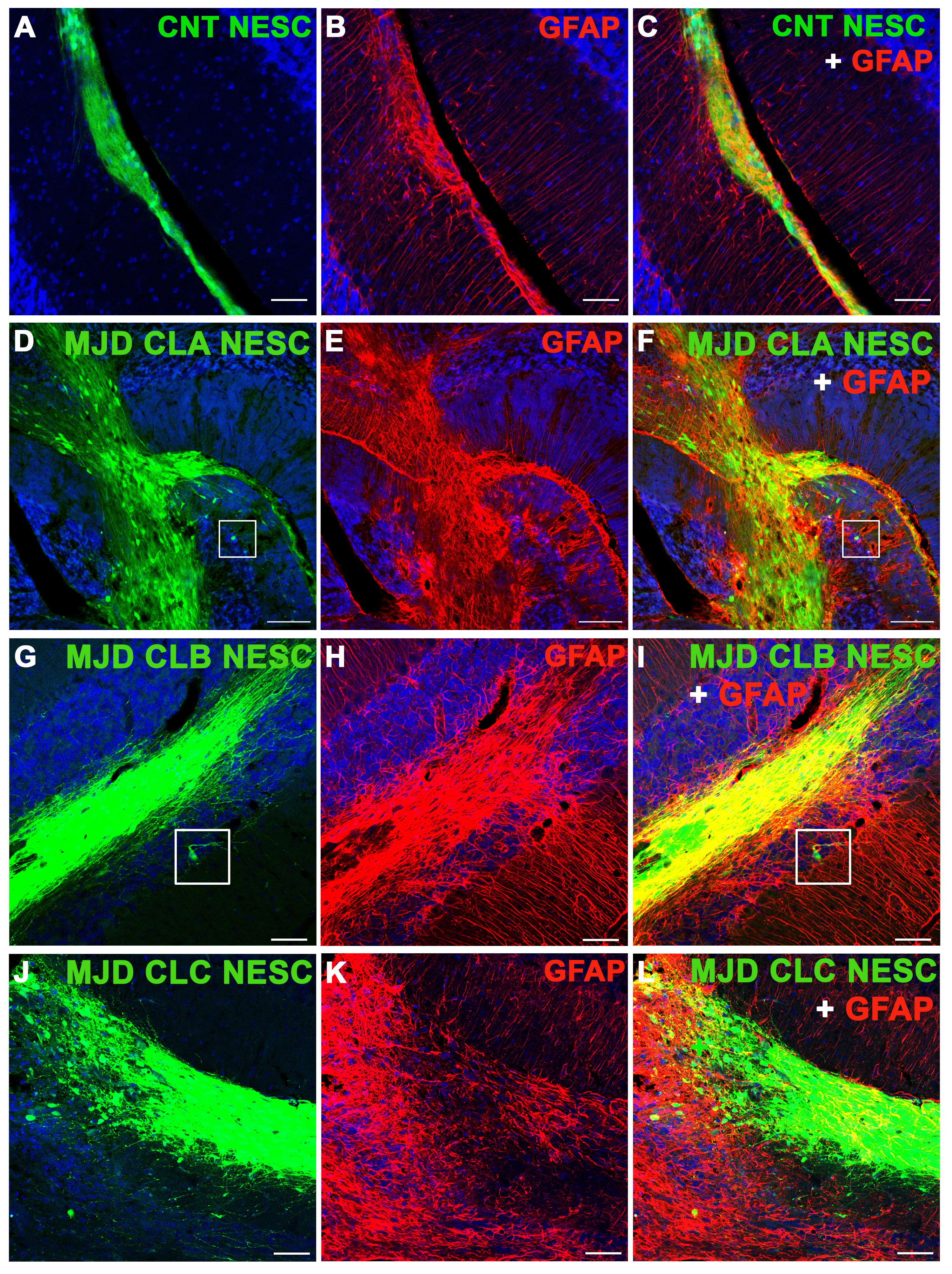


**Supplementary Fig. S5.** **Differentiation of Control and MJD iPSC-derived NESC into glia in the cerebellum of adult mice.** Representative fluorescence confocal microscopy images of GFAP immunolabeling in NOD.SCID mice cerebellar sections two months after cell transplantation. Data show that **(A-C)** Control (CNT NESC), **(D-F)** MJD CLA (MJD CLA NESC), **(G-I)** MJD CLB (MJD CLB NESC), and **(J-L)** MJD CLC (MJD CLC NESC) iPSC-derived NESC expressing GFP (green) differentiate into GFAP-positive (red) glial cells showed by the colocalization between GFP and GFAP. White squares indicate the presence of graft-derived cells in the Purkinje cell layer. DAPI: blue, n=3 mice/group, Scale bars: 100 μm.


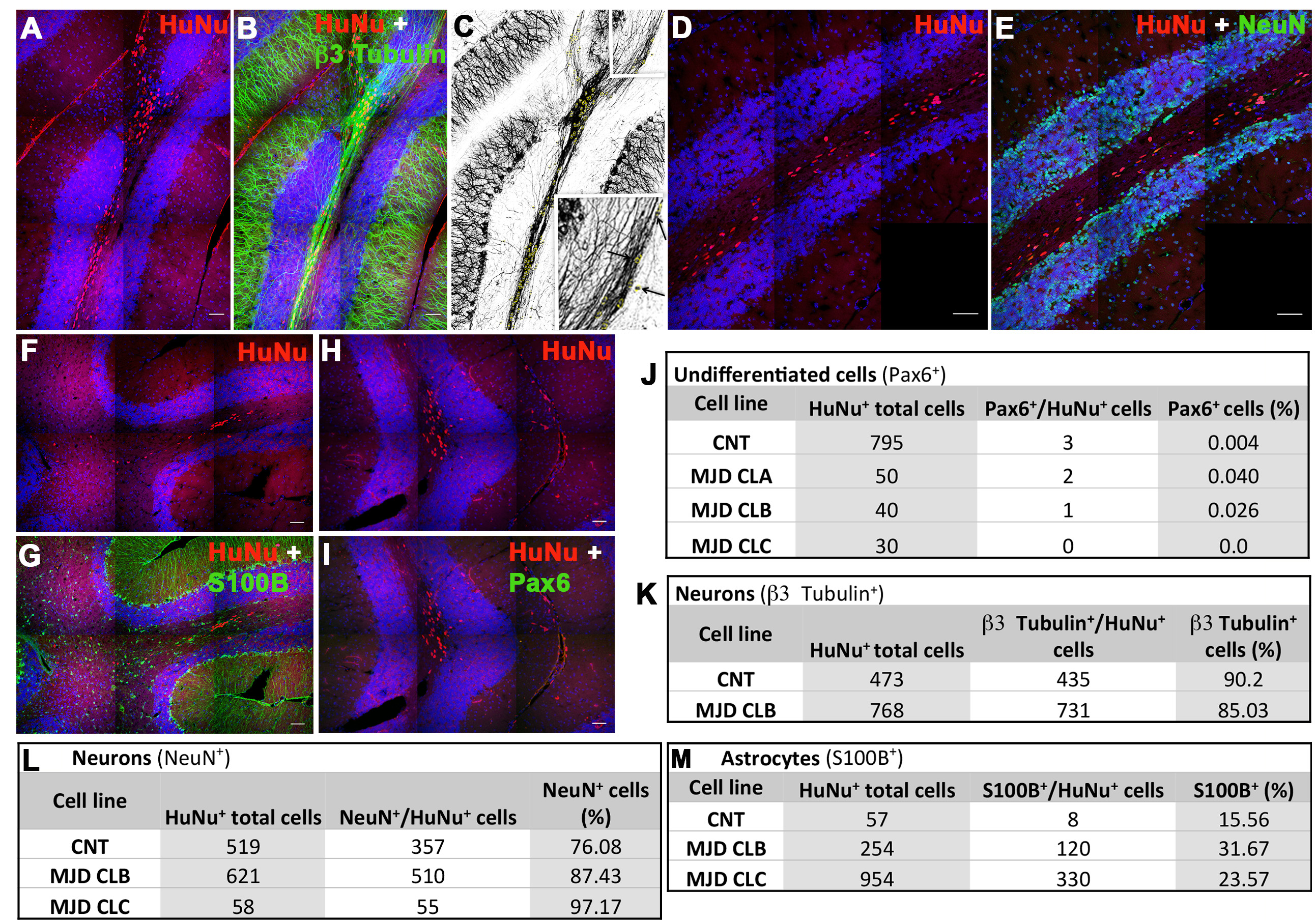


**Supplementary Fig. S6. *In vivo* differentiation of human iPSC-derived NESC in neurons and glia.** Differentiation of human iPSC-derived NESC assessed by colocalization of human nuclei antigen (HuNu) with neuronal markers (β3 Tubulin and NeuN, green), astrocytes (S100B, green), and undifferentiated cells (Pax6, green) in cerebellar sections of mice transplanted with the human cells. Representative immunofluorescence confocal microscopy images showing human iPSC-derived NESC differentiated in (**A-E**) neurons and (**F-G**) astrocytes and (**H-I**) undifferentiated cells. **C**) Representative image of orthogonal reconstruction of the confocal z-stack pictures showing HuNu and β3 Tubulin colocalization. Number and percentage of Control (CNT), MJD CLA, MJD CLB, and MJD CLC iPSC-derived NESC (**J**) undifferentiated (Pax6^+^/HuNu^+^ cells) and differentiated in neurons (**K**) (β3 Tubulin^+^/ HuNu^+^ cells) and (**L**) (NeuN^+^/ HuNu^+^ cells), and (**M**) astrocytes (S100B^+^/ HuNu^+^ cells) in the human grafts (HuNu^+^ total cells) at 2 months post-transplantation. J) CNT, CLA and CLC n= 1 and CLB n=2 mice/group; K) n= 2 mice/group; L) CNT and CLC n= 2 and CLB n=3 mice/group; M) CNT n= 1 and CLB and CLC n=2 mice/group. Scale bars: 50 μm.


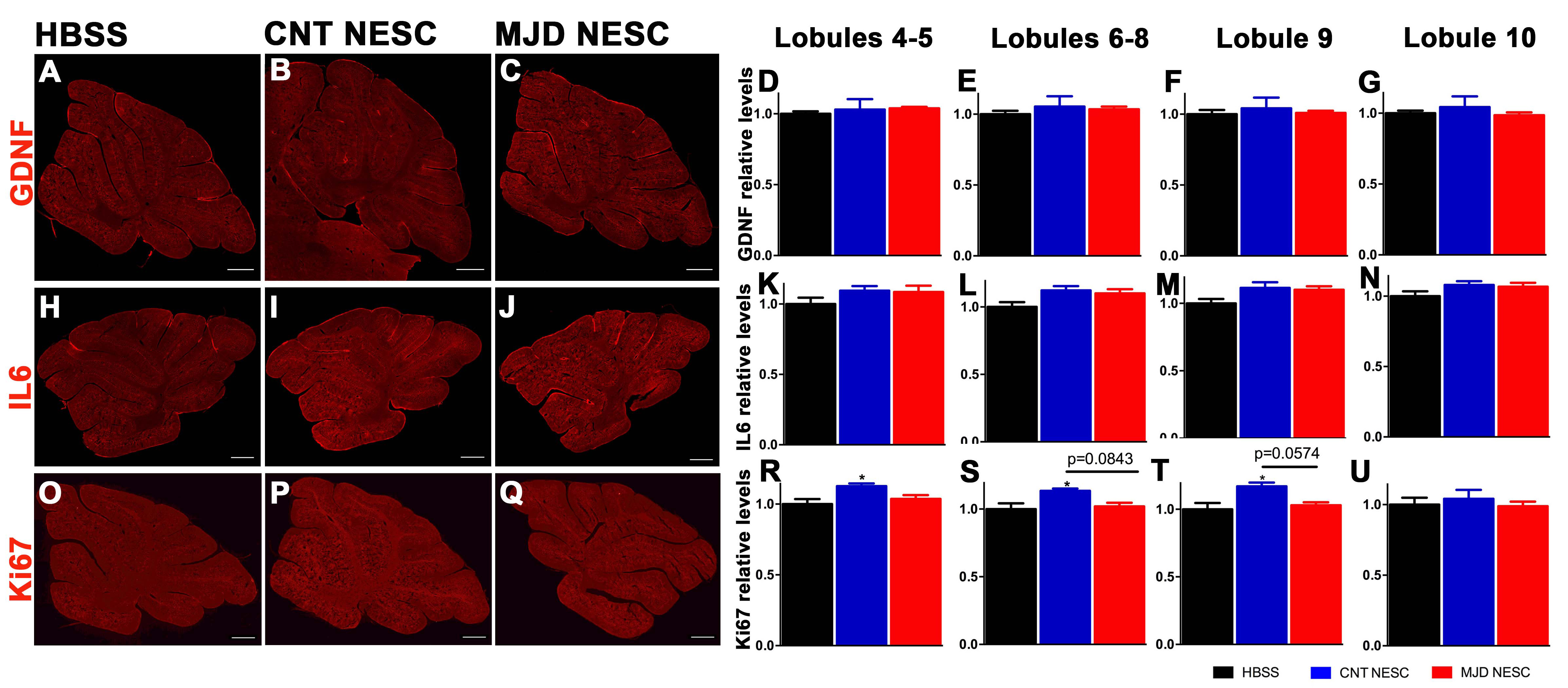


**Supplementary Fig. S7. Control and MJD CLA iPSC-derived NESC transplantation impact in GDNF, IL6, and Ki67 cerebellar levels.** Six months after cerebellar transplantation of Control (CNT NESC) and MJD CLA (MJD NESC) iPSC-derived NESC expressing GFP in adult NOD.SCID mice it was measured the cerebellar levels of the neurotrophic factor GDNF, pro-inflammatory interleukin IL6, and cell proliferation marker Ki67. Representative immunofluorescence images of **(A-C)** GDNF, **(H-J)** IL6, and **(O-Q)** Ki67 labeling in the cerebellum of mice injected with (**A**, **H**, **O**) HBSS and transplanted with (**B**, **I**, **P**) CNT and (**C**, **J**, **Q**) MJD iPSC-derived NESC; scale bars: 500 μm. Levels of **(D-G)** GDNF, **(K-N)** IL6, and **(R-U)** Ki67 in cerebellar lobules 4 and 5 (Lobules 4-5), 6 to 8 (Lobules 6-8), 9 (Lobule 9), and 10 (Lobule 10) measured through fluorescence medium intensity normalized for the HBSS group. HBSS n=4, NESC CNT n=4, NESC MJD n=3. Data are expressed as mean ± SEM, **p*<0.05, One-way ANOVA followed by Tukey’s post-test.


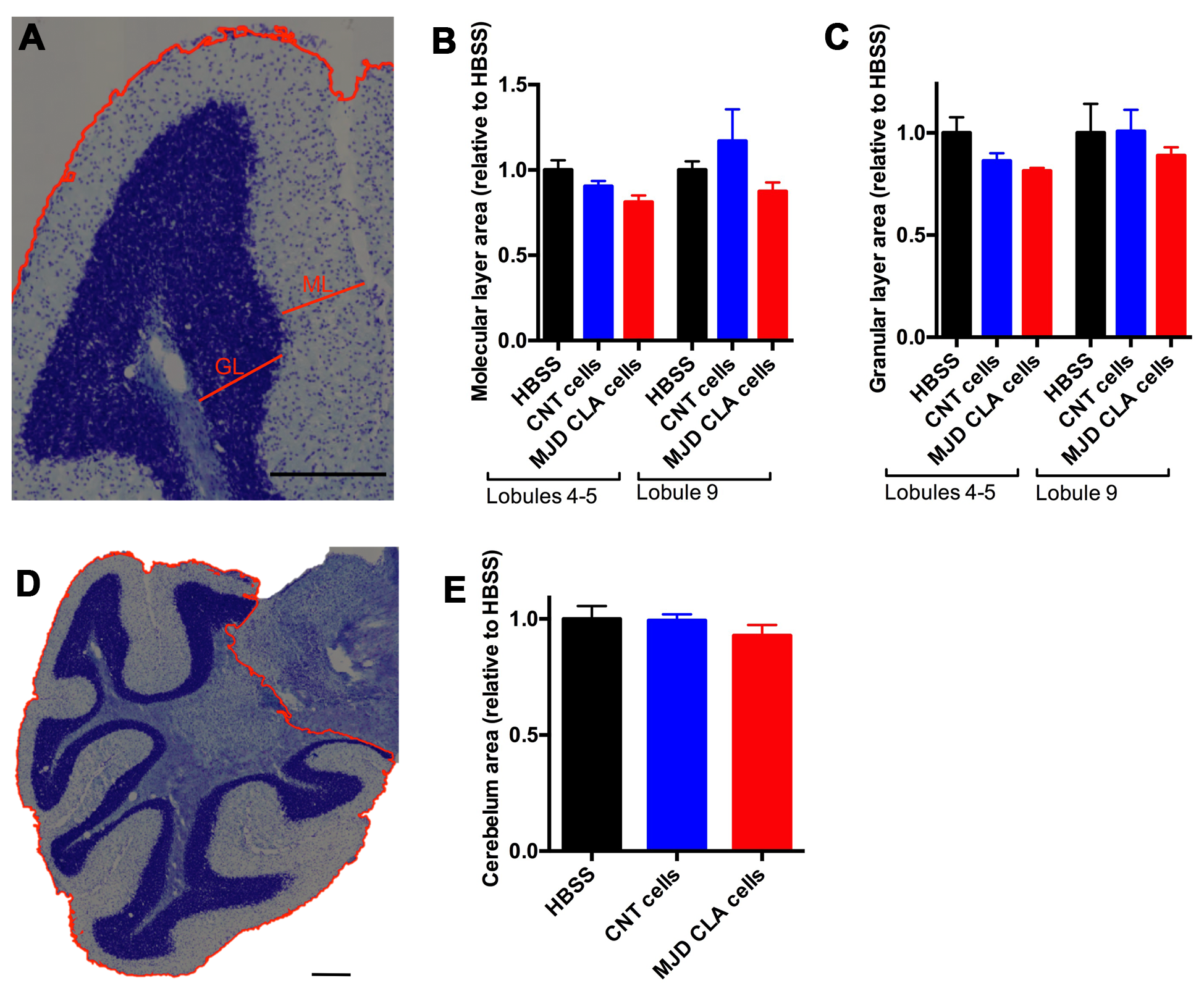


**Supplementary Fig. S8. Human iPSC-derived NESC grafts promoted no significant changes in cerebellar cellular layers area and cerebellar total area. A**) Representative image of Cresyl violet staining of molecular (ML) and granular (GL) layers in cerebellar sections, scale bar: 250 μm. **B-C**) ML and GL area in lobules 4-5 (cell graft zone) and lobule 9 (far from the cell graft zone) of mice transplanted with CNT iPSC-derived NESC (CNT cells) and MJD iPSC-derived NESC (MJD CLA cells), normalized to the control (HBSS) mice, revealed no significant differences compared with control mice injected with saline solution (HBSS). **D**) Representative image of Cresyl violet staining of whole cerebellum, scale bar = 500 μm. **E**) Whole cerebellum area in control mice (HBSS) and in mice transplanted with human iPSC-derived NESC (CNT cells and MJD CLA cells), normalized to the control mice, revealed no significant impact in cerebella area by the transplantation of human cells. CNT cells and HBSS: n=4 mice and MJD CLA cells: n=3 mice. Data are expressed as mean ± SEM, One-way ANOVA followed by Tukey’s post-test.


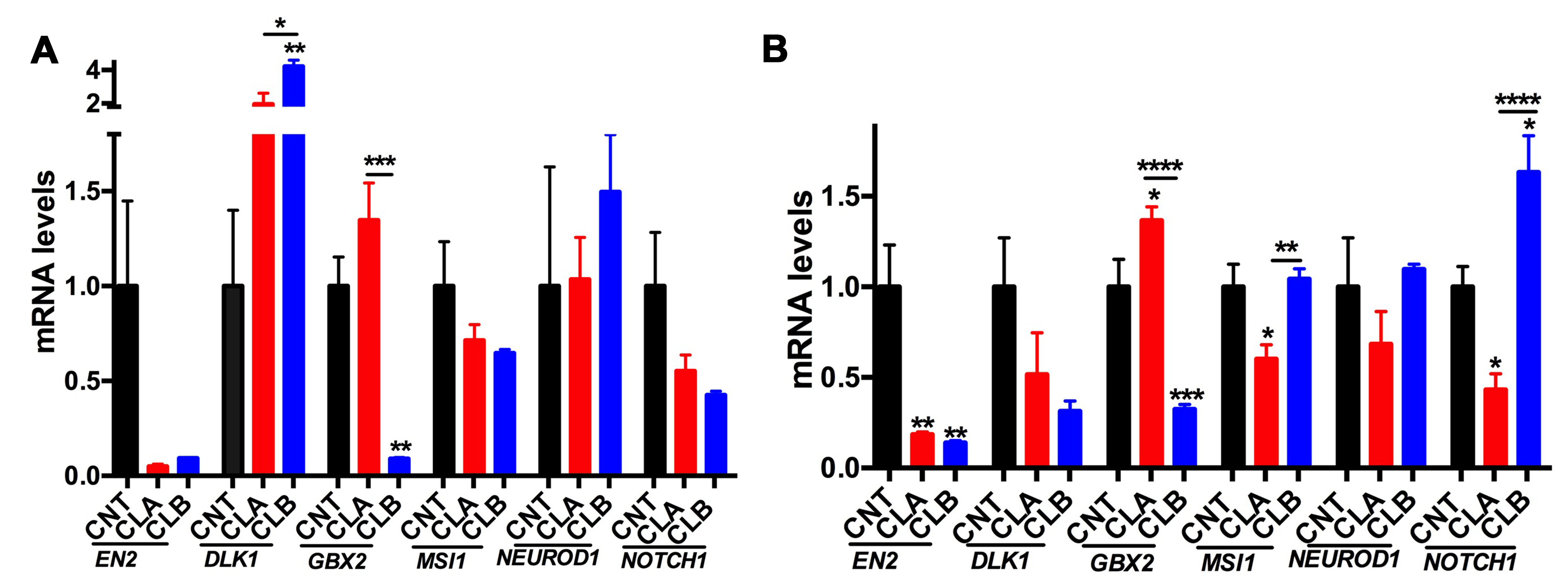


**Supplementary Fig. S9. Potential therapeutic potency predictive genes mRNA levels evaluation.** *EN2*, *DLK1*, *GBX2*, *MSI1*, *NEUROD1*, and *NOTCH1* mRNA levels were evaluated through RT-qPCR in **(A)** CNT, MJD CLA (CLA), and MJD CLB (CLB) iPSC-derived NESC and in **(B)** their respective cell cultures differentiated for 3 days. Data revealed higher *EN2*, *MSI1*, and *NOTCH1* mRNA levels in CNT differentiated cell cultures; n=5-7 independent experiments. Data are expressed as mean ± SEM, **p*<0.05, ***p*<0.01, ****p*<0.001, *****p*<0.0001, One-way ANOVA followed by Tukey’s post-test.


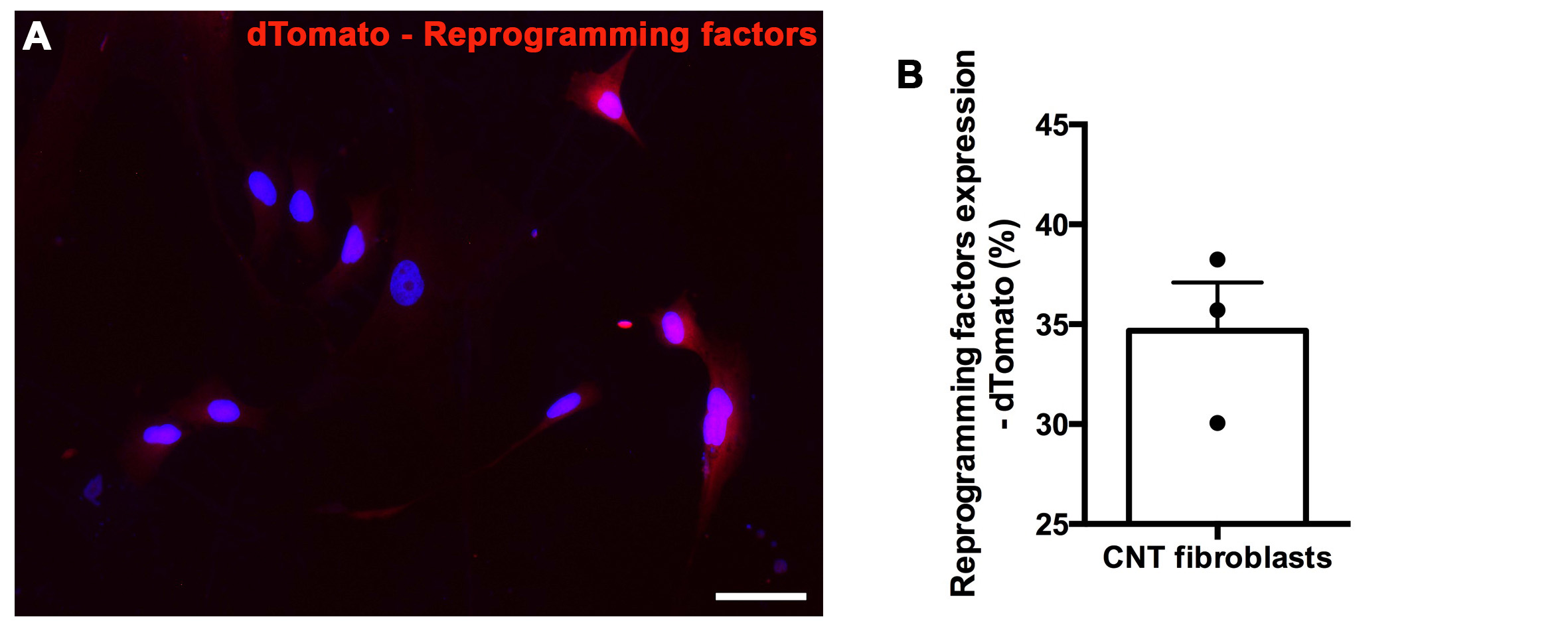
**Supplementary Fig. S10. Transduction of human fibroblasts with cell reprogramming factors.** **A**) Representative fluorescence microscopy image showing human CNT fibroblasts transduced with lentivirus expressing the 4 reprogramming factors (c-Myc, Klf4, Sox2, and Oct4) and the dTomato fluorescent protein (red), DAPI = blue, scale bar = 50 μm. **B**) Quantification of fibroblasts expressing dTomato four days after transduction revealed that 34.67% ± 4.19 of fibroblasts express the cell reprogramming factors; n = 3 independent experiments.

**Western blot membranes of Figure 1:**


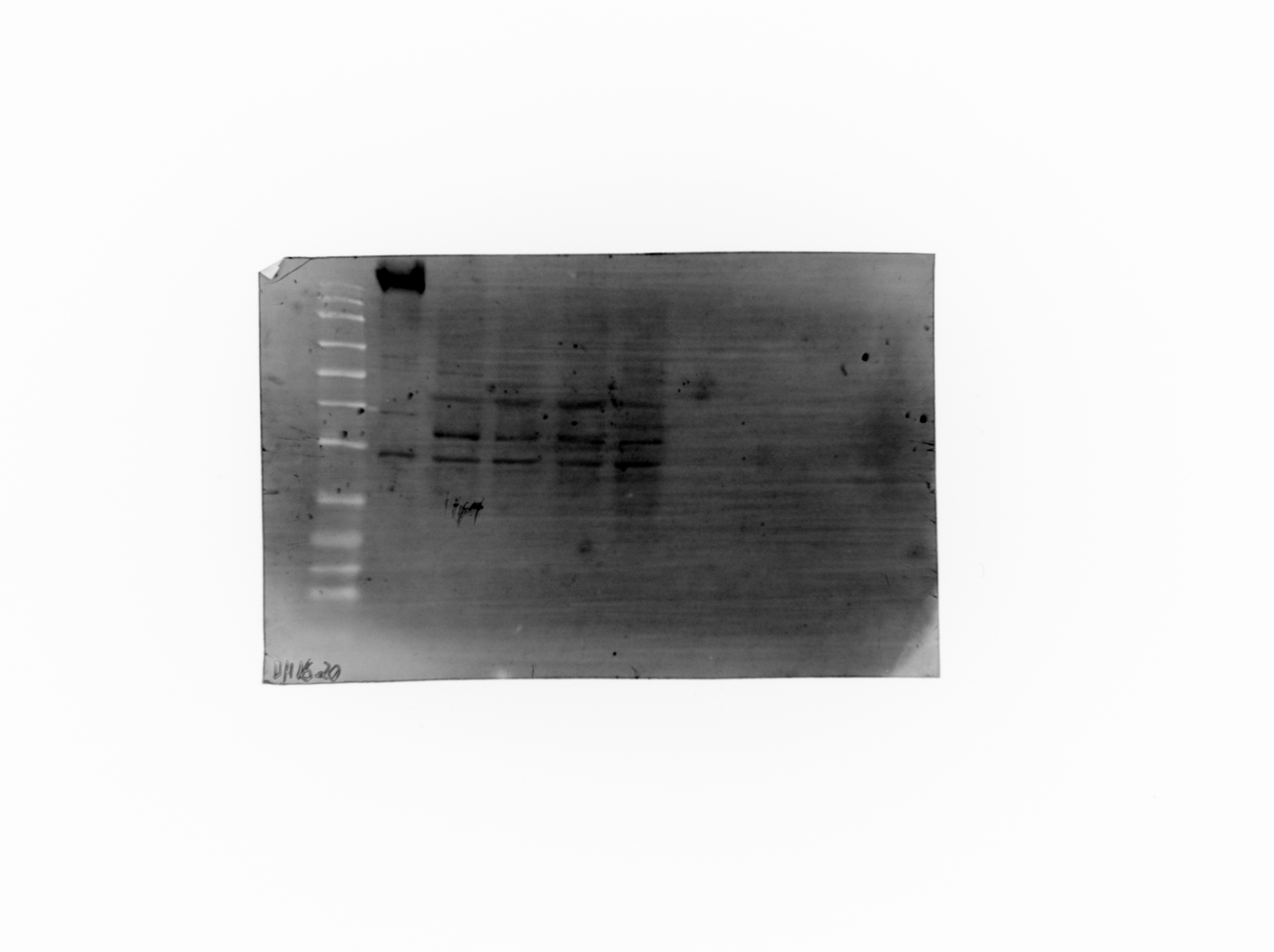


**MJD iPSC**

**CNT NESC**

**MJD CLA NESC**

**MJD CLB NESC**

**MJD CLC NESC**

**Tra-1-60**

235 KDa


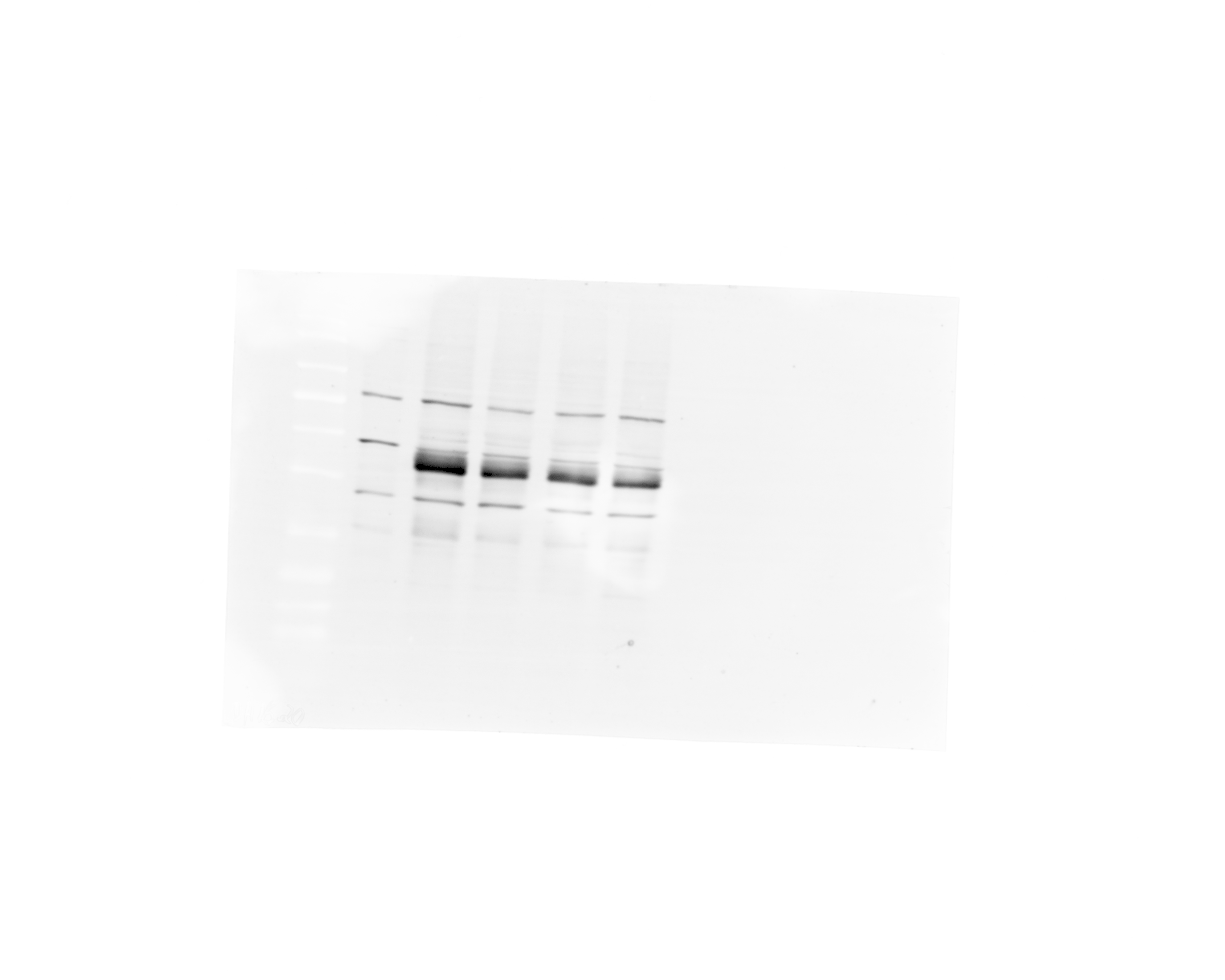


**Pax6**

47KDa

**MJD iPSC**

**CNT NESC**

**MJD CLA NESC**

**MJD CLB NESC**

**MJD CLC NESC**


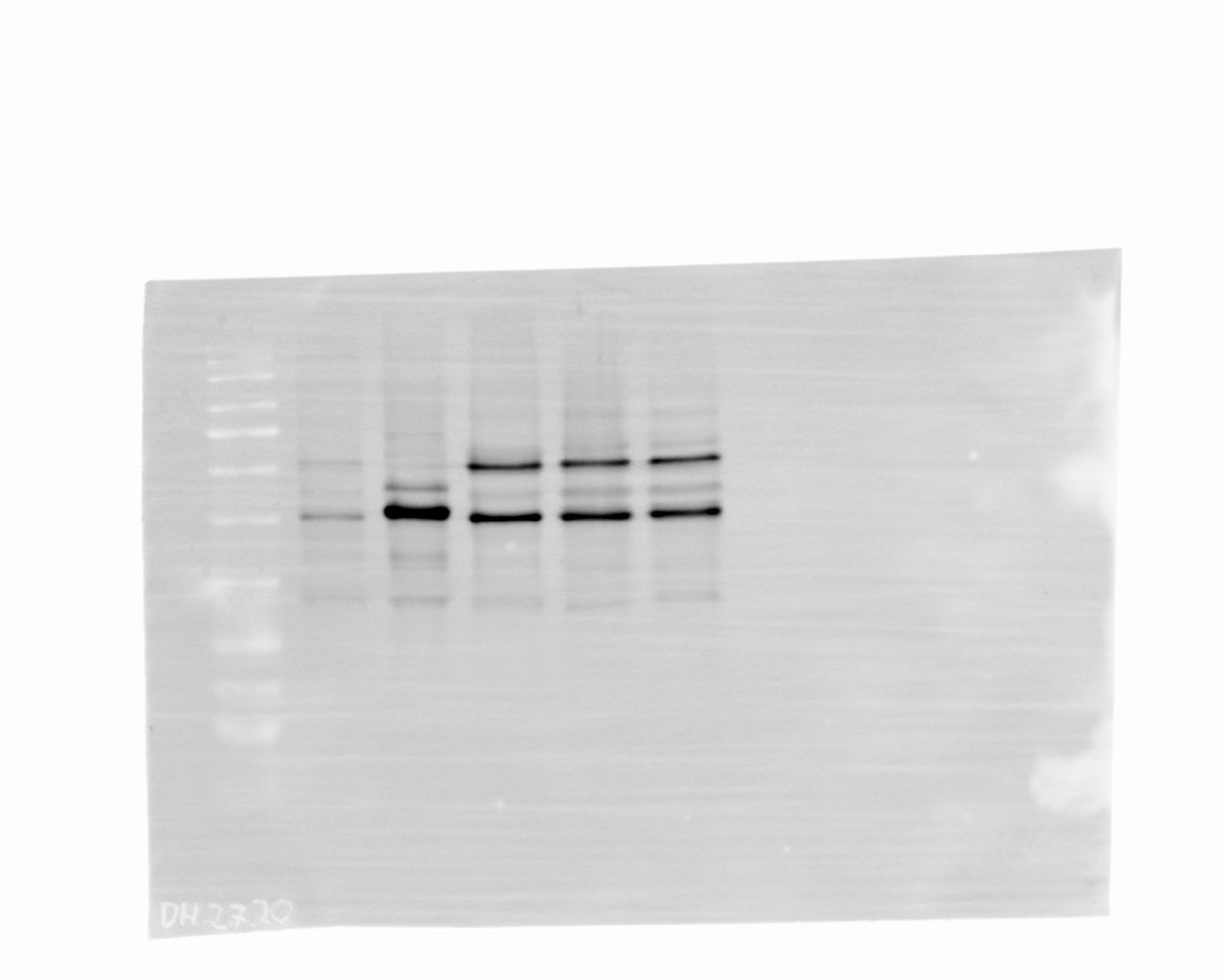


**Mutant ataxin-3**

67 KDa

**Wild-type ataxin-3**

50 KDa

**MJD iPSC**

**CNT NESC**

**MJD CLA NESC**

**MJD CLB NESC**

**MJD CLC NESC**

**Actin**

42 KDa

**MJD iPSC**

**CNT NESC**

**MJD CLA NESC**

**MJD CLB NESC**

**MJD CLC NESC**


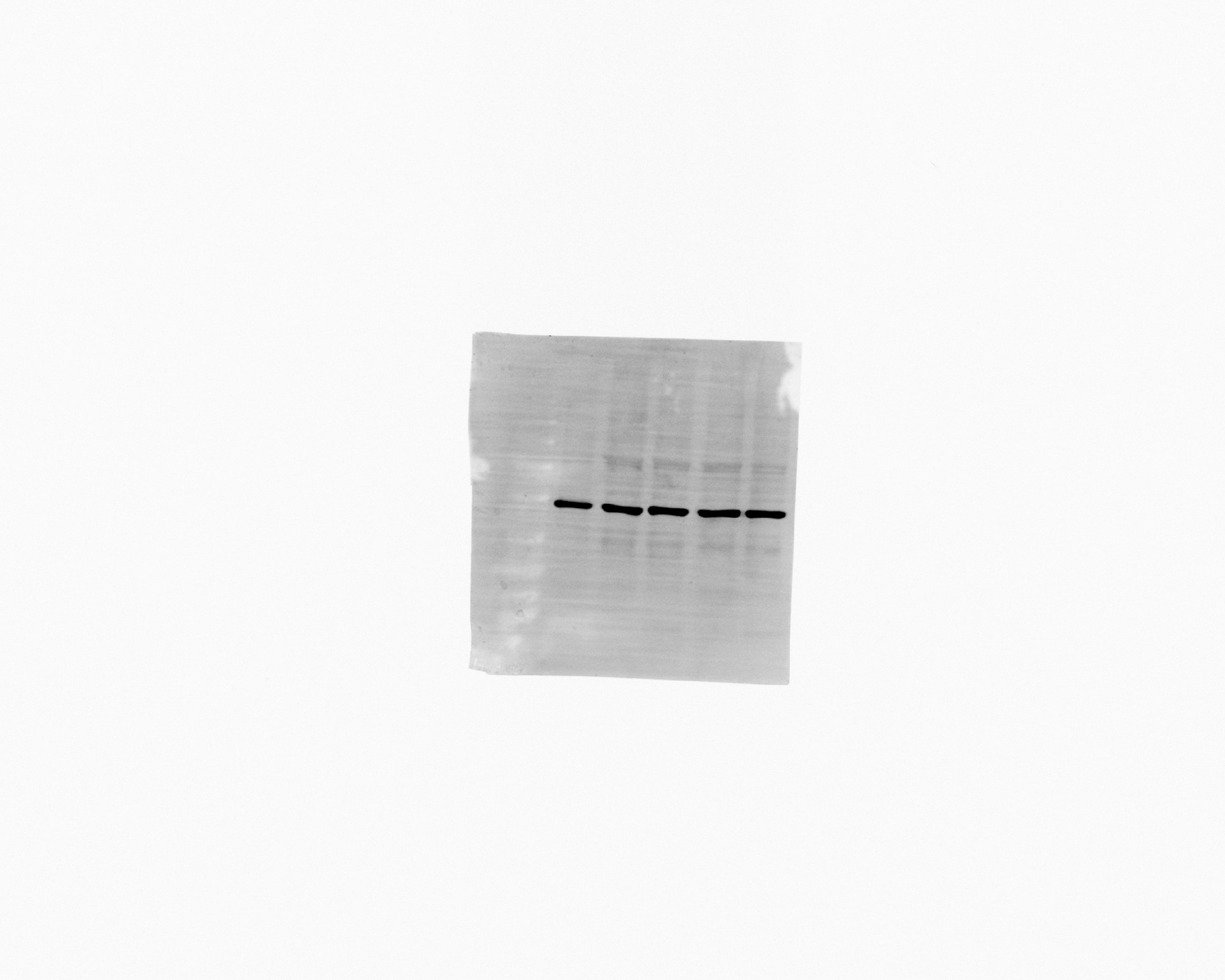

Supplement: Supplementary file 1 — Supplementary Information. [file 41598_2024_53542_MOESM1_ESM.docx]
